# A data-driven computational model for obesity-driven diabetes onset and remission through weight loss

**DOI:** 10.1016/j.isci.2023.108324

**Published:** 2023-10-23

**Authors:** Vehpi Yildirim, Vivek M. Sheraton, Ruud Brands, Loes Crielaard, Rick Quax, Natal A.W. van Riel, Karien Stronks, Mary Nicolaou, Peter M.A. Sloot

**Affiliations:** 1 Department of Public and Occupational Health, Amsterdam University Medical Centers, University of Amsterdam, 1081 BT Amsterdam, the Netherlands; 2 Institute for Advanced Study, University of Amsterdam, 1012 GC Amsterdam, the Netherlands; 3 Computational Science Lab, University of Amsterdam, 1098 XH Amsterdam, the Netherlands; 4 Center for Experimental and Molecular Medicine, Amsterdam University Medical Centers, 1100 DD Amsterdam, the Netherlands; 5 AMRIF B.V., Agro Business Park, 6708 PW Wageningen, the Netherlands; 6 Institute for Risk Assessment Sciences, Utrecht University, 3584 CL Utrecht, the Netherlands; 7 Department of Biomedical Engineering, Eindhoven University of Technology, 5600 MB Eindhoven, the Netherlands; 8 Department of Experimental and Vascular Medicine, Amsterdam University Medical Centers, 1100 DD Amsterdam, the Netherlands

**Keywords:** Human metabolism, Bioinformatics, Computational bioinformatics

## Abstract

Obesity is a major risk factor for the development of type 2 diabetes (T2D), where a sustained weight loss may result in T2D remission in individuals with obesity. To design effective and feasible intervention strategies to prevent or reverse T2D, it is imperative to study the progression of T2D and remission together. Unfortunately, this is not possible through experimental and observational studies. To address this issue, we introduce a data-driven computational model and use human data to investigate the progression of T2D with obesity and remission through weight loss on the same timeline. We identify thresholds for the emergence of T2D and necessary conditions for remission. We explain why remission is only possible within a window of opportunity and the way that window depends on the progression history of T2D, individual’s metabolic state, and calorie restrictions. These findings can help to optimize therapeutic intervention strategies for T2D prevention or treatment.

## Highlights

• A data-driven model for obesity-driven progression of diabetes and remission
• The model successfully reproduces clinical trial data for diabetes remission
• The model identifies a window of opportunity for remission through weight loss
• These findings could help optimize diabetes prevention and treatment strategies

## Graphical abstract

**Figure:**
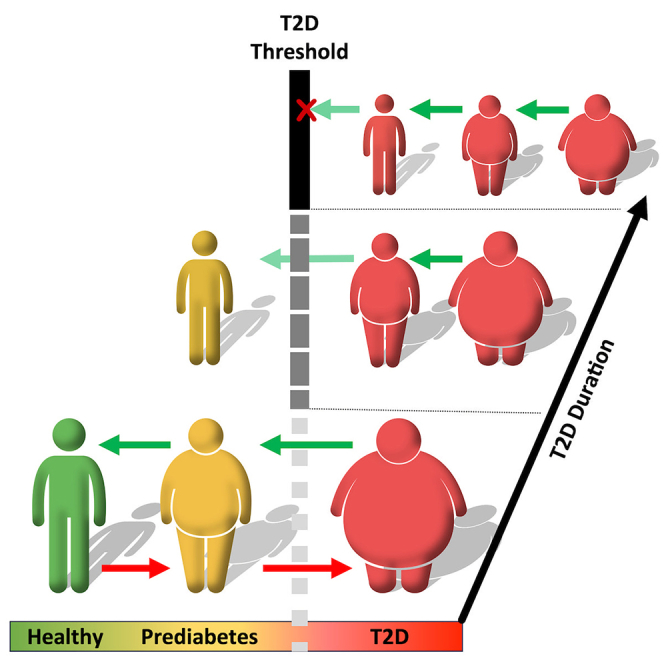

## Introduction

Obesity is one of the major risk factors for the development of insulin resistance and type 2 diabetes (T2D) and a frequent comorbidity seen in these individuals.^1^ In obesity, ectopic fat deposition in liver and muscle impairs insulin signaling in these cells and results in insulin resistance.^2^^,^^3^^,^^4^ Elevated intracellular lipids and their byproducts can also cause pancreatic β-cell dysfunction and impair insulin secretion.^5^ Another factor that links obesity to T2D is inflammation.^6^^,^^7^ In obesity, elevated levels of proinflammatory mediators impair insulin signaling in liver, muscle, and adipose tissue cells^8^^,^^9^^,^^10^ and cause β-cell dysfunction as well as diminish insulin secretion.^11^^,^^12^^,^^13^

In individuals with obesity and T2D, a substantial and sustained weight loss can result in T2D remission, through improved insulin sensitivity and β-cell recovery.^14^^,^^15^^,^^16^^,^^17^^,^^18^^,^^19^ Similar improvements in T2D indices and remission are also reported in bariatric surgery patients.^20^^,^^21^^,^^22^^,^^23^ In individuals with obesity and T2D, all weight loss interventions result in improved metabolic health,^24^^,^^25^ but remission takes place only in a fraction of these individuals.^15^^,^^26^ Studies suggest that the extent of the weight loss, the duration of the T2D, and the overall metabolic and medical state of the individual might be important determinants for the success of remission with weight loss.^14^^,^^16^^,^^18^^,^^19^^,^^26^ Therefore, to develop sustained (therapeutic and environmental) intervention strategies for prevention or reversal of T2D in individuals with obesity, it is imperative to study the progression history of T2D and remission process together. Although experimental and observational studies provide information regarding the difference between the metabolic variables at the healthy state, during T2D, and after remission, they cannot explain the dynamics of the transition of an individual from one state to another. Furthermore, these studies do not account for the dynamic interactions between metabolic variables during the progression of the disease, as they are limited to snapshots of a number of variables at a few time points. These limitations arise from the slow progression of T2D, its multifactorial nature, and metabolic heterogeneity among individuals with obesity.^27^^,^^28^ Hence, these studies do not allow for investigating the progression of diabetes with weight gain and remission through weight loss together. In contrast, computational modeling provides the opportunity to study the obesity-driven progression of T2D and remission on a single timeline in the same individual. Computational models can keep track of several metabolic variables and their interactions continuously and identify important points in disease progression. Moreover, models can be used to analyze *what-if* scenarios and make future predictions, where extrapolation of information from experimental data is not possible.

In the past, several computational models have been proposed to describe the dynamics of T2D progression.^29^^,^^30^^,^^31^^,^^32^^,^^33^^,^^34^^,^^35^^,^^36^^,^^37^ They provide valuable information regarding the progression of T2D in relation to impaired insulin sensitivity and a progressive decline in β-cell mass and constitute a solid basis for the current study. However, these models do not include the physiological mechanisms underlying the decline in insulin sensitivity and its relation to body weight; insulin sensitivity is modeled either as a simple control parameter or as a decreasing function of time, where it starts to decline at an arbitrary setoff point in life without any physiological cue or regulator.^29^^,^^31^^,^^32^^,^^35^^,^^36^ Although this approximation is desirable to simply investigate the effect of a decline in insulin sensitivity on long-term glycemic control and β-cell mass, it cannot account for the multifactorial interplay between obesity, insulin resistance, and impaired insulin secretion during the development of T2D. Furthermore, although some of these models include body weight as a parameter,^33^^,^^36^ none of them include weight gain dynamics. Therefore, they can explain neither the dynamic relationship between weight gain and the progression of T2D nor the effect of the weight loss on T2D remission.

We address these issues by developing a computational model that describes the progression of T2D with weight gain and remission through weight loss. The model accounts for the dynamic interactions between plasma glucose, insulin, free fatty acids (FFA), insulin sensitivity, systemic inflammatory status, β-cell function, and β-cell mass in relation to weight gain and increased adiposity over years. The model can simulate the effects of different weight gain and weight loss regiments on the emergence of T2D and remission. To this end, we use previously published diabetes remission clinical trial (DIRECT) study data to calibrate and validate the model.^38^ Our results show that the model can successfully reproduce experimental and observational findings and provide deeper insights into the underlying physiological mechanisms.

## Results

**Figure 1:**
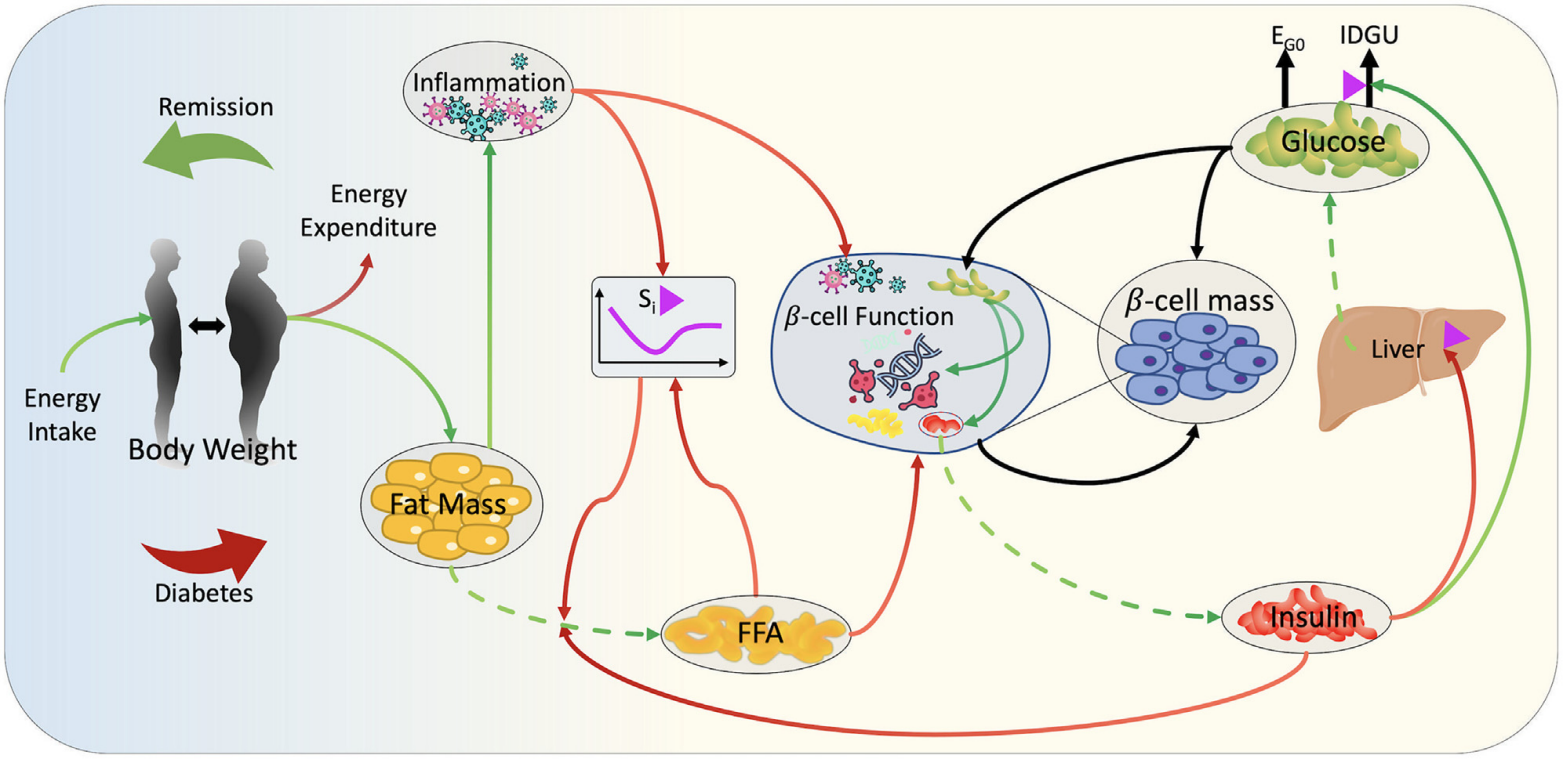
The model diagram Green arrows represent positive relations, whereas red arrows indicate negative relations. The black arrows represent non-monotonic relations, where the polarity depends on the state of the variables. The magenta triangles indicate that the process depends on insulin sensitivity. Dashed arrows indicate production and secretion of metabolites or hormones, whereas solid arrows indicate a regulatory effect. S_i_, insulin sensitivity; EGP, endogenous glucose production; E_G0_, insulin-independent glucose uptake; IDGU, insulin-dependent glucose uptake; FFA, free fatty acids.

The interactions between the model components are summarized in Figure 1, and their details are provided in the STAR Methods section. The model describes the dynamic changes in glucose, insulin, free fatty acids (FFA), and inflammation levels in relation to weight gain and their roles in the progression of insulin resistance and T2D. In the model, T2D emerges as a result of insulin resistance in combination with insufficient insulin secretion, which is secondary to impaired β-cell function and reduced β-cell mass. In short, weight gain and resulting increase in adipose tissue mass leads to increased plasma FFA and systemic inflammation. Plasma FFA increases ectopic fat accumulation and induces insulin resistance in peripheral tissues and causes β-cell dysfunction. Inflammation also impairs peripheral insulin sensitivity and β-cell function. Consequently, reduced β-cell function results in a decline in insulin secretion, which, together with reduced insulin sensitivity, leads to an increased glucose production in liver (HGP), a reduced glucose uptake (IDGU), and a further increase in FFA release from adipose tissue. As a result, reduced insulin sensitivity and insulin levels lead to hyperglycemia that pushes β-cell metabolic rate to its limits. This high metabolic demand leads to a further decline in β-cell function and eventually results in β-cell death. At the baseline, we assume that the system is in a metabolically healthy state (for definition of metabolic states see STAR Methods), where the total daily energy intake and total daily energy expenditure are in balance, body mass index (BMI) is maintained at 25 kg/m^2^, and plasma glucose levels are below the prediabetes cutoff, which is set to 100 mg/dL. In the model, a perpetual imbalance between energy intake and energy expenditure results in weight gain that initiates a chain of events that pushes the system out of the healthy state and results in insulin resistance and T2D (Figure 1).

### The emergence of T2D depends on the extent of weight gain

**Figure 2:**
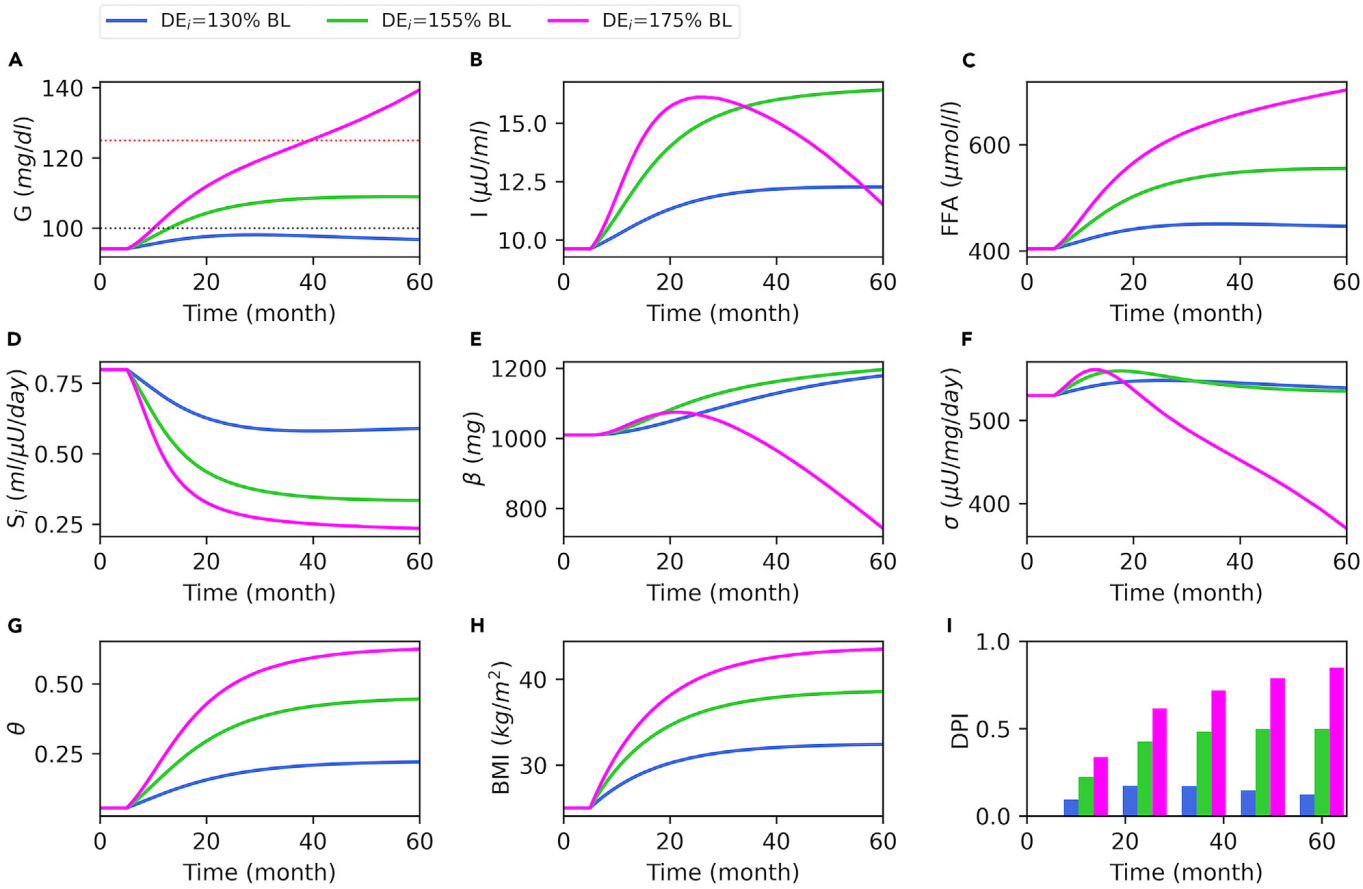
Model simulations for different levels of increase in daily energy intake (*DE*_*i*_) At T = 5 months, *DE*_*i*_ is increased to 130% (blue), 155% (green), or 175% (magenta) of the baseline (BL). Plasma glucose-*G* (A), insulin-*I* (B), FFA (C), insulin sensitivity-*S*_*i*_ (D), β-cell mass-*β* (E), β-cell function-*σ* (F), inflammation index-*θ* (G), and BMI (H) are shown for a time course of 60 months. The T2D progression index (DPI) is shown for every 12 months (I). In panel A, the black and red dotted lines at 100 *mg/dL* and 125 *mg/dL* mark the prediabetes and T2D cutoffs for plasma glucose level, respectively.

To understand the relationship between weight gain and the progression of insulin resistance and T2D, model simulations were generated under different weight gain scenarios over a time course of 60 months (Figure 2) using the parameter values given in Table S3. Simulations start at a healthy state, and weight gain is initiated by increasing total daily energy intake (*DE*_*i*_) over the baseline value (BL) at T = 5 months.

Increasing DE_i_ to 130% of the baseline (BL) increases BMI to over 30 kg/m^2^ within two years (Figure 2H, blue), which slightly increases *FFA* (Figure 2C, blue) and the systemic inflammation index (*θ*) (Figure 2G, blue) over their respective baselines. Increased *FFA* and *θ* reduce insulin sensitivity (*S*_*i*_) by nearly 25% (Figure 2D, blue) and cause an increase in plasma glucose (*G*) (Figure 2A, blue). Concurrently, β-cell function (σ) starts to increase (Figure 2F, blue) in response to elevated glucose, and it is followed by β-cell mass (*β*) (Figure 2E, blue) on a slower timescale. The increase in *σ* and *β* compensates for the reduction in *S*_*i*_ by increasing plasma insulin (*I*) (Figure 2B, blue), which keeps glucose under the prediabetes level (Figure 2A, black dotted line). In this scenario, elevated σ and β together compensate for the reduced insulin sensitivity and keep plasma glucose under control, by increasing insulin secretion.

**Figure 3:**
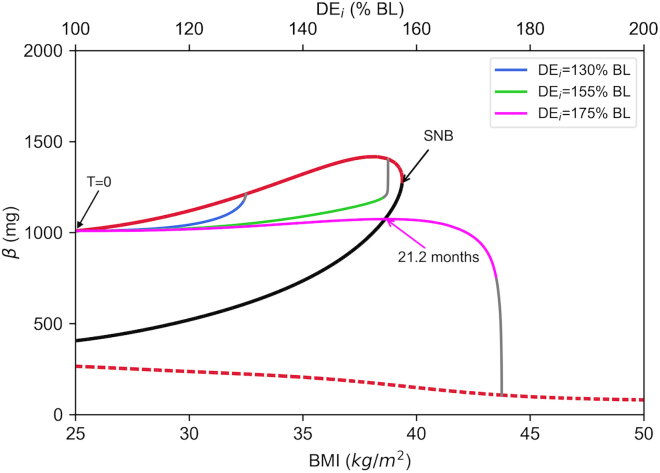
Bifurcation diagram of the dynamical system Each point on this bifurcation diagram represents a steady state for *β* versus a corresponding BMI value. The steady state solutions of the system are projected onto the BMI-*β* axes. Solid and dashed red curves show the branches of upper and lower stable steady states, respectively, whereas the solid black curve shows the branch of unstable steady states. The superimposed trajectories are the projection of the solution curves given in Figure 2. T = 0 points to the initial state or the baseline for the trajectories, where the gray segments represent the portion of the corresponding solution curve that exceeds 60 months. The arrow labeled with 21.2 months indicates the point at which magenta solution takes its maximal *β* value in months (Figure 2E, magenta), which coincides with the branch of unstable steady states. DE_i_ relative to the baseline (% BL) necessary for corresponding steady state BMI values are labeled on the top axis. SNB, saddle node bifurcation.

Increasing *DE*_*i*_ to 155% of the baseline causes a more pronounced increase in *BMI* (Figure 2H, green), followed by a substantial increase in *FFA* (Figure 2C, green) and *θ* (Figure 2G, green). Consequently, *S*_*i*_ reduces considerably (Figure 2D, green), which increases glucose to the prediabetes ranges (Figure 2A, green). In green simulations, where *DE*_*i*_ is increased to 155% of the baseline, *σ* is initially increased (Figure 2F, green), but after a while it starts to decline as glucose and FFA reach the cytotoxic levels (Figures 2A and 2C). At this point, the increase in *β* is (Figure 2E, green) sufficient to maintain high insulin levels that keep glucose under the T2D level (Figure 2A, red dotted line). Finally, increasing *DE*_*i*_ to 175% of the baseline causes a significant weight gain in a relatively short period of time (Figure 2H, magenta). This results in high *FFA* (Figure 2C, magenta) and *θ* (Figure 3G, magenta), which reduces *S*_*i*_ by more than 75% (Figure 2D, magenta). There is an initial increase in *σ* and *β* (Figures 2E and 2F, magenta), but this is not sufficient to keep glucose under control. Consequently, first *σ* starts to decline due to the severe glucolipotoxicity. The decline in *σ* reduces the insulin secretion rate (*ISR*), which further increases glucose levels and β-cell metabolic rate (*M*). Consequently, *β* starts to decline (Figure 2E, magenta), and T2D emerges. During the progression into the diabetic state, insulin follows a nonmonotonic time course (Figure 2B, magenta), whereas glucose levels progressively increase (Figure 2A, magenta), and hyperglycemia concurrently exists with hyperinsulinemia for several years.

Our model simulations show that a decline in *S*_*i*_ is sufficient to raise glucose to the prediabetes levels but not to the diabetic levels (Figure S3, blue), where a substantial decline in β-cell function and β-cell mass is necessary for the development of T2D (Figure S3, magenta). Therefore, we define a T2D progression index (*DPI*) as a function of *S*_*i*_, *σ*, and *β* to summarize the overall diabetes state (Figure 2I, see STAR Methods for details). In summary, the decline in insulin sensitivity due to the low or moderate weight gain can be compensated by increased β-cell function and β-cell mass and resulting hyperinsulinemia, whereas a more pronounced increase in body weight cannot be compensated and results in T2D. Although these simulations show that T2D emerges at BMI levels above 40 kg/m^2^, depending on the level of inflammation and other metabolic variables, T2D can emerge at lower BMI (see later discussion).

### System dynamics exhibit bistability, where β-cell mass and BMI define a threshold for the emergence of T2D

Figure 2 shows that weight gain results in T2D, as a consequence of reduced *S*_*i*_, *σ*, and *β.* Moreover, there seems to be a weight gain threshold that determines the emergence of T2D. In order to understand the long-term behavior of the system and investigate the weight gain threshold for the emergence of T2D, we focus on the steady state solutions and the slow dynamics of the system. For this purpose, we perform a continuation analysis and generate a bifurcation diagram of the system using BMI as the bifurcation parameter. The resulting bifurcation diagram is a representation of the steady state solutions of the system for a range of BMI values in 8-dimensional space (see Figure S5 in supplemental information and accompanying text for details). Figure 3 shows the projection of the 8-dimensional bifurcation diagram onto the *BMI-β*-axes. Due to the large difference between the timescales of *β* and other system variables (Figures S4 and S5 in supplemental information), this projection allows us to accurately analyze the slow dynamics and the long-term behavior of the system.

In Figure 3, we also superimpose three solution trajectories that correspond to the time courses shown in Figure 2 to draw a more dynamic picture. The diagram shows a bistable region for BMI values between 25 and 39.5 kg/m^2^, with 2 sets of stable steady state solutions (stable nodes, Figure 3, solid and dashed red curves) and a set of unstable steady state solutions (saddle points, Figure 3, bold black curve). There is a limit point, where the branch of upper stable steady states coalesces with the branch of unstable steady states and disappears at a saddle node bifurcation (Figure 3, SNB). In the bistable region in Figure 3, the branch of unstable solutions separates the basin of attraction for trajectories and constitutes a threshold for the emergence of T2D. On the left side of the branch of unstable steady states, the solutions are attracted by the high *β* stable steady states (Figure 3, solid red curve). On the right side of the branch of unstable steady states, the solutions are attracted by the low *β* stable steady states (Figure 3, dashed red curve). Therefore, if weight gain pushes the phase point to the right side of the branch of unstable steady states, *β* converges to the corresponding lower stable steady state and T2D emerges. In contrast, if the trajectory remains on the left side of the branch of unstable steady states (or is pushed to the left side of the threshold by a weight loss intervention), the phase point is attracted by the high *β* stable steady states and individual does not become diabetic (or achieves remission, which is discussed in detail in the following section).

Increasing DE_i_ from the baseline causes an increase in BMI through weight gain, so the phase points move to the right (Figure 3, blue, green, and magenta). The phase point refers to the state of the system in 8-dimensional phase-space of model variables, and its flow generates the solution trajectories. Increasing DE_i_ by 55% increases BMI to 38 kg/m^2^ but this does not push the phase point beyond the threshold (Figure 3, green). Therefore, the solution is attracted by the high *β*-cell stable steady state. However, increasing DE_i_ by 75% causes BMI to rise above 43 kg/m^2^, which pushes the phase point beyond the threshold, where it is attracted by the low *β* stable steady state (Figure 3, magenta). In Figure 3, the magenta trajectory starts off by moving to the right and upward with increased BMI until it reaches the threshold, because it is inside the basin of attraction of the high *β* stable steady states. As the phase point passes beyond the threshold, it enters the basin of attraction of the low *β* stable steady states, and it starts moving downward. Consequently, *β* follows a nonmonotonic time course and takes its maximal value at 21.2 months (Figure 2E, magenta). This time point corresponds to the point where the magenta trajectory passes through the branch of unstable steady states in Figure 3. In Figure 2, at 21.2 months, the magenta simulations exhibit insulin resistance with moderate hyperglycemia, but T2D has not emerged yet. However, the analysis in Figure 3 reveals that, after 21.2 months, it is already evident that the magenta simulation will converge to a low *β* steady state and T2D will emerge.

### There exists a window of opportunity for remission through weight loss, which depends on T2D duration, β-cell mass, BMI, and the intensity of the calorie restriction at the intervention time point

**Figure 4:**
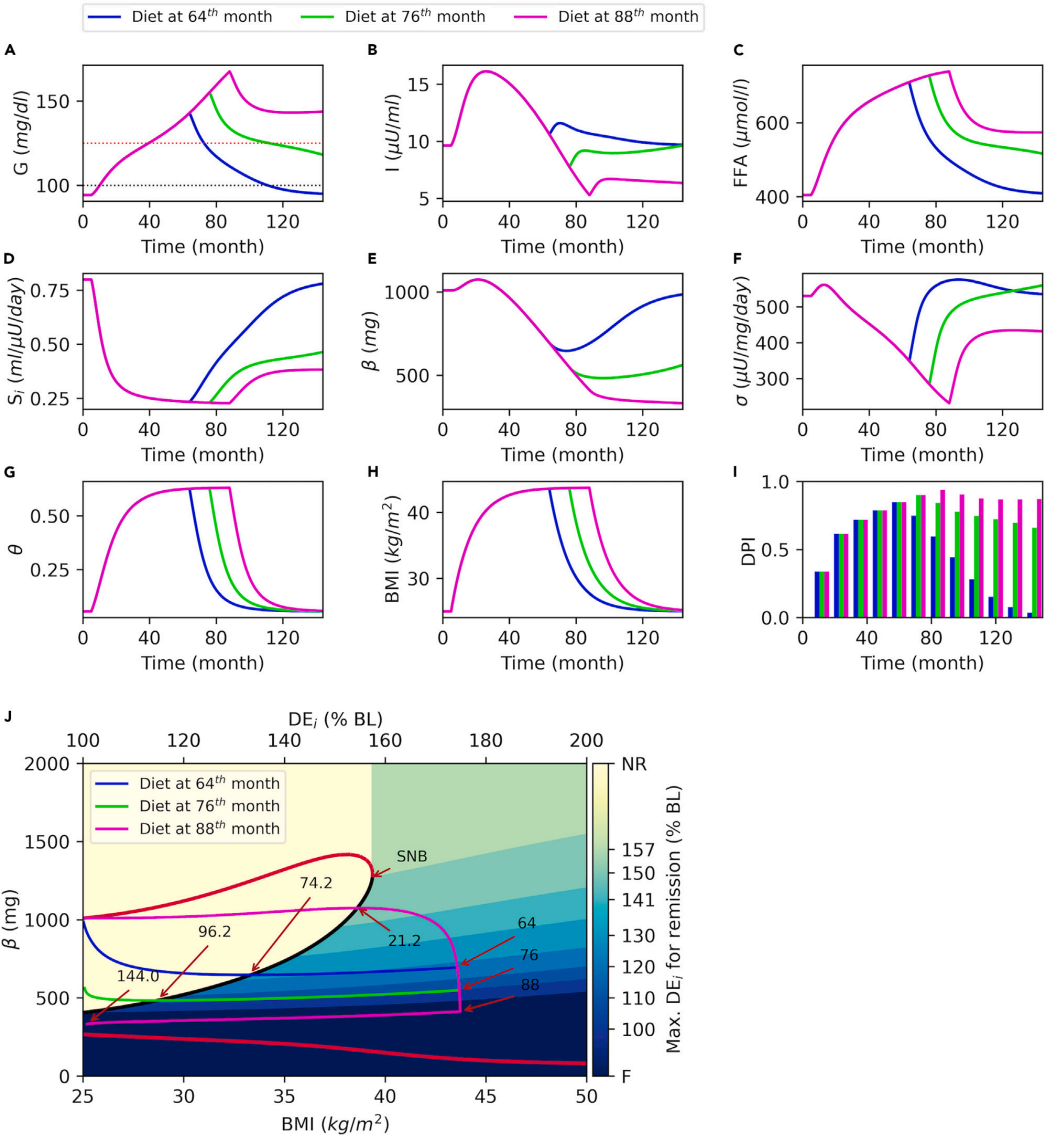
Diabetes remission through weight loss At T = 5 months, daily energy intake (*DE*_*i*_) is increased to 175% of the baseline (BL) to simulate weight gain. To simulate diet interventions, *DE*_*i*_ is set back to the baseline value at T = 64 (blue), 76 (green), or 88 months (magenta). (A) Plasma glucose with prediabetes (black dotted line, 100 *mg/dL*) and T2D cutoffs (red dotted line, 125 *mg/dL*). (B) Plasma insulin, (C) plasma FFA, (D) insulin sensitivity, (E) β-cell mass, (F) β-cell function, (G) inflammation index, and (H) BMI time courses. (I) Disease progression index (DPI) at every 12 months. (J) The projection of the bifurcation diagram onto *BMI-β* axes with superimposed trajectories. The brown arrows are used to label the time points of certain events in months (*β-*maximum, intervention, and *β-*minimum). *DE*_*i*_ relative to baseline for the corresponding steady state BMI values are labeled on the top axis. The color bar shows the maximum *DE*_*i*_ levels relative to baseline (% BL) necessary for successful remission within corresponding region. BL, baseline; NR, no restriction; F, failure; SNB, saddle node bifurcation.

To investigate the impact of weight loss on the reversal of T2D symptoms and remission, we simulate weight gain scenarios followed by diet interventions at different time points following T2D onset. In Figure 4, at T = 5 months, DE_i_ is increased from baseline by 75%, which increases BMI to more than 42 kg/m^2^ (Figure 4G) and raises glucose to the T2D levels (Figure 4A) within 3 years (at around T = 40 months). To understand the effect of diet interventions that take place at different points in time, DE_i_ is set back to the baseline level at T = 64 (Figure 4, blue), 76 (Figure 4, green), and 88 months (Figure 4, magenta).

The intervention that takes place nearly 2 years after T2D onset successfully reduces glucose below the prediabetes ranges (Figure 4A, blue). The intervention results in a significant weight loss, which is followed by reduced FFA (Figure 4C, blue) and inflammation levels (Figure 4G, blue). As a consequence, S_i_ increases to its baseline within the simulation time frame (12 years). *σ* and *β* also rise back to their respective baseline values (Figures 4E and 4F, blue). Hence, DPI drops to zero by the end of the simulation (Figure 4I, blue), indicating a complete recovery. The diet intervention that takes place nearly 3 years after T2D onset results in a similar weight loss (Figure 4, green). Although plasma glucose drops below the T2D levels, it remains within the prediabetes range with a decreasing trend (Figure 4A, green). After the intervention, inflammation returns to its baseline level (Figure 4G, green) following the BMI’s time course, but FFA does not (Figure 4C, green). This is because a feedback loop exists among FFA, insulin, and *S*_*i*_, which makes FFA dependent on *σ* and *β*. As a result of the feedback between *S*_*i*_ and FFA, *S*_*i*_ also does not improve as much as it does in blue simulations after the intervention (Figure 4D, compare green with blue). In green scenario, *σ* rises nearly back to its baseline levels within the first 12 months following the intervention (Figure 4F, green), but *β* increases very slowly (Figure 4E, green). Nevertheless, despite the limited increase in *β*-cell mass, elevations in *σ* and S_i_ are sufficient to induce remission by reducing plasma glucose below the T2D levels (Figures 4A and 4I, green). On the other hand, the intervention that takes effect at T = 88 months (nearly 4 years after the disease onset) does not result in remission. Although S_i_ (Figure 4D, magenta) and *σ* are significantly improved, *β* does not increase at all. In fact, the decline in *β* continues at a slower pace. Therefore, there is only a moderate decline in plasma glucose (Figure 4A, magenta), which remains in the T2D range.

In all three scenarios presented in Figure 4, the diet interventions are initiated at the same BMI value (Figure 4H), and more importantly, all interventions result in the same BMI at the end of the simulations. However, each intervention results in a different metabolic outcome. To elucidate this discrepancy, which is solely caused by the time of the interventions, we investigate the solution curves presented in Figures 4A–4G, against the bifurcation diagram introduced in Figure 3. For this purpose, we superimpose solution trajectories onto the bifurcation diagram (Figure 4J). The phase points start off at the baseline BMI and *β* values (25 kg/m^2^, 1000 mg). As weight gain progresses, initially, all the solutions move to the right and upward until they reach the branch of unstable steady states that serves as a threshold. At T = 21.2 months, as the trajectories pass through the threshold, they continue moving right but they turn downward, because they are attracted by the lower stable steady states. Notice that the trajectories pass through the threshold at 21.2 months (Figure 4J), where the plasma glucose levels are still below the T2D range in Figure 4A. This implies that the decline in β-cell mass starts earlier than the onset of T2D. However, the decline in β-cell mass is very slow compared with the increase in BMI. As the phase point approaches 43.75 kg/m^2^ on the BMI axes, which is the steady state BMI level that results from 75% increase in DE_i_, the rightward movement in the BMI direction slows down and the downward movement starts dominating.

The diet interventions are initiated at T = 64, 76, and 88 months, where the trajectories make sharp leftward turns due to the reduced BMI (Figure 4J). After the intervention, trajectories move left and downward until they reach the threshold. Therefore, the decline in *β* continues after the intervention until the phase point reaches the threshold (Figures 4E and 4J), where they turn upward, and *β* starts increasing. Notice that trajectories that start moving leftward at higher *β* levels (or in this setup earlier in time), need to travel a shorter distance to reach the threshold. Indeed, after the intervention, the blue trajectory passes through the threshold in 10 months and starts moving upward at a relatively higher BMI (33.3 kg/m^2^), whereas it takes 20 months for the green trajectory to reach the threshold at a lower BMI (28.7 kg/m^2^). This result indicates that recovery in *β*-cell mass could be significantly faster if the intervention takes place earlier (Figure 4J), showing the importance of early intervention. On the other hand, the magenta trajectory cannot reach the threshold at all, and remission fails. This is because at the point the magenta trajectory reaches the baseline BMI level, it ends up below the threshold and is attracted by the lower *β* stable steady state.

From this we can conclude that remission is possible if the weight loss intervention takes place within a window of opportunity after the T2D onset. The intensity of the diet intervention necessary for a successful remission depends on BMI values and *β*-cell mass at the intervention time point. In Figure 4J, the intensity of the calorie restriction necessary for successful remission is color coded. In the yellow region at the top left corner (NR), no restriction is necessary because, in this region, solutions are already attracted by the high *β* stable steady states. In contrast, in the dark blue region at the bottom, remission is not possible at all, even if the DE_i_ is set back to the baseline. In the top right region, the color gradient from green to blue represents the DE_i_ necessary for successful remission relative to the baseline level (% BL). For instance, if the trajectory reaches (or if it is initiated from) the region marked by 120%, DE_i_ must be set below 120% of the baseline for a successful remission (Figure S3). Another important finding is that the longer the individual remains in the diabetic state, the further the *β* will decline, and the trajectory will reach the darker regions on that map (Figure 4J). This implies that, at the earlier stages of the T2D, a moderate diet intervention might be sufficient for remission, whereas the longer the individual remains in the diabetic state the more intensive the diet intervention becomes necessary for remission (Figure S6). The upper boundary of the dark blue region in Figure 4J defines a threshold, above which the remission is possible with the right diet intervention at the right time. This boundary starts off at nearly 400 mg *β*-cell mass (40% of the baseline) and stretches almost linearly to the 540 mg *β*-cell mass with increased BMI. In other words, the window of opportunity for remission shrinks with increased BMI.

Our model also shows that prevention of T2D is metabolically less challenging than the remission (Figure S7). Furthermore, after a successful remission, due to the slow recovery of the β-cell mass, T2D can easily relapse with weight gain (Figures S7J and S7K). This result shows that individuals who achieve remission through weight loss are more likely to develop T2D with weight regain, compared with the weight-matched non-diabetic individuals. Our model also predicts that fast weight gain is more detrimental for glucose metabolism than weight gain at a slower pace (Figure S8), because *β*-cell function and mass cannot adapt to the changes induced by a fast weight gain.

### Inflammation aggravates the negative effects of weight gain, accelerates the progression of T2D, and reduces the remission success

**Figure 5:**
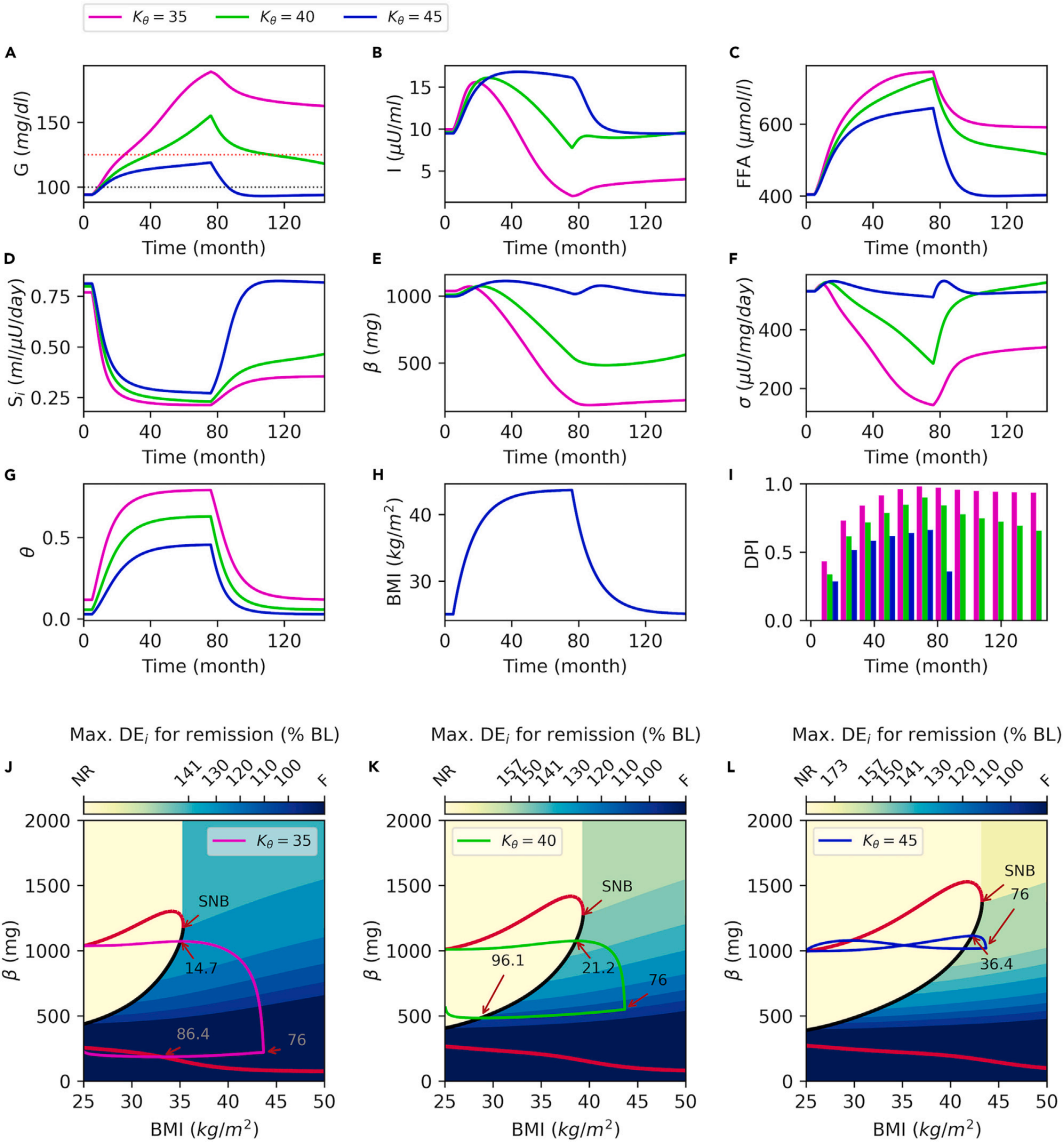
Effects of inflammation on diabetes progression and remission Model simulations for different values of the parameter *K*_*θ*_, which determines the inflammatory response to increased BMI. (A) Plasma glucose with prediabetes (black dotted line, 100 *mg/dL*) and T2D cutoffs (red dotted line, 125 *mg/dL*). (B) Plasma insulin, (C) plasma FFA, (D) insulin sensitivity, (E) β-cell mass, (F) β-cell function, (G) inflammation index, and (H) BMI time courses. (I) Disease progression index (DPI) at every 12 months. (J–L) The projection of the bifurcation diagram onto *BMI-β* axes with superimposed trajectories for corresponding *K*_*θ*_ values. The color bar shows the maximum *DE*_*i*_ levels relative to baseline (% BL) necessary for successful remission for a corresponding region. BL, baseline; NR, no restriction; F, failure; SNB, saddle node bifurcation.

In order to understand the effect of obesity-driven inflammation on the progression of T2D and remission, we investigated the impact of elevated inflammatory responses on the system dynamics. In the model, inflammation negatively affects *S*_*i*_ and *σ*. The model represents the overall inflammation level by an inflammation index (*θ*), which dynamically changes in response to BMI. In the model, *K*_*θ*_ determines the intensity of the inflammatory responses to BMI by shifting the response curve to the right (Equation 11). Hence, a lower *K*_*θ*_ value corresponds to a stronger inflammatory response to BMI and higher inflammation levels. In Figure 5, at T = 5 months, DE_i_ is increased by 75% over the baseline for different *K*_*θ*_ values, and at T = 76 months, DE_i_ is reduced to the baseline to simulate a diet intervention scenario.

In each scenario, BMI follows the same time course (Figure 5H) but other metabolic responses are completely different because higher inflammation levels cause a greater decline in *S*_*i*_ and *σ*. This in turn results in a lower insulin level (Figure 5B) and a higher glucose concentration (Figure 5A). Also, notice that FFA follows different time courses, despite the same BMI (Figure 5H) and associated fat mass. Because, in the model, adipose tissue lipolysis and FFA production depend on insulin and *S*_*i*_. Therefore, lower insulin levels and lower *S*_*i*_ caused by higher inflammation levels result in higher FFA (Figure 5C). Together these cause a significantly low *σ* and *β* (Figures 5E and 5F) and a greater disease progression (Figure 5I). Weight gain with low inflammation (Figure 5G, blue) leads to prediabetes but it does not result in T2D (Figure 5A, blue). Furthermore, metabolic variables quickly return to their respective baselines after the weight loss, indicating a full recovery (Figures 5A–5H, blue). On the other hand, weight gain with moderate or high inflammation (Figure 5G, green and magenta) results in T2D (Figure 5A, green and magenta). However, only in the case of moderate inflammation level does the weight loss result in remission (Figure 5A, green). With high inflammation, weight loss results in improved *S*_*i*_ (Figure 5D, magenta) and *σ* (Figure 5F, magenta) but *β* does not increase (Figure 5E, magenta), and remission fails (Figure 5A, magenta).

Figure 5 shows that inflammatory responses have a significant impact on the emergence of T2D and remission. In order to investigate these effects, once again we turn our attention to the steady state solution of the system. Figures 5J–5L show the projected bifurcation diagram of the system for different *K*_*θ*_ values along with corresponding solution trajectories shown in Figures 5A–5H. For this analysis, we use *K*_*θ*_ = 40 kg/m^2^ as the reference value (which is the nominal value used in the model) and compare the effects of higher and lower values of *K*_*θ*_. For higher values of *K*_*θ*_, which corresponds to lower inflammation, the bifurcation diagram is shifted to the right and stretched in *β*-axis (Figures 5L compared with 5K). Consequently, the trajectory passes through the branch of unstable steady states that serves as a threshold later in time and at higher BMI values (Figures 5K–5L, 21.2 versus 36.4 months). Moreover, after the phase point passes through the threshold, it does not move too far to the right and remains in the vicinity of the unstable steady state, where the flow in *β* direction is very slow. Consequently, at the intervention time point (76 months), *β* does not decline much. In this region, even a small calorie restriction would be sufficient for remission (reducing DE_i_ from 175% of the baseline to 157% of the baseline). Therefore, reducing DE_i_ to the baseline results in complete recovery.

On the other hand, for lower values of *K*_*θ*_, the diagram is shifted to the left and shrunken in *β*-axis (Figures 5J compared with 5K). Hence, a lower BMI value is sufficient to push the phase point beyond the threshold. Consequently, the trajectory passes through the threshold earlier in time at lower BMI (Figure 5L, 14.7 versus 21.2 moths). After the phase point passes through the threshold, it moves farther to the right, where the flow in the *β* direction is faster. Consequently, at the intervention time point (76 months), *β* is already declined to the dark blue region, where weight loss would not succeed in remission. Notice that for lower values of *K*_*θ*_ and higher inflammation (Figure 5J), the region where remission is possible becomes darker, which implies more drastic calorie restrictions for remission. On the other hand, for higher values of *K*_*θ*_ (Figure 5L), the region where remission is possible becomes lighter, which implies a lower calorie restriction for remission. In summary, inflammation accelerates the emergence of T2D at lower BMI. Moreover, it is more challenging to resolve T2D that emerges with high inflammation.

### Diabetes emerges through different metabolic profiles at similar BMI values

**Figure 6:**
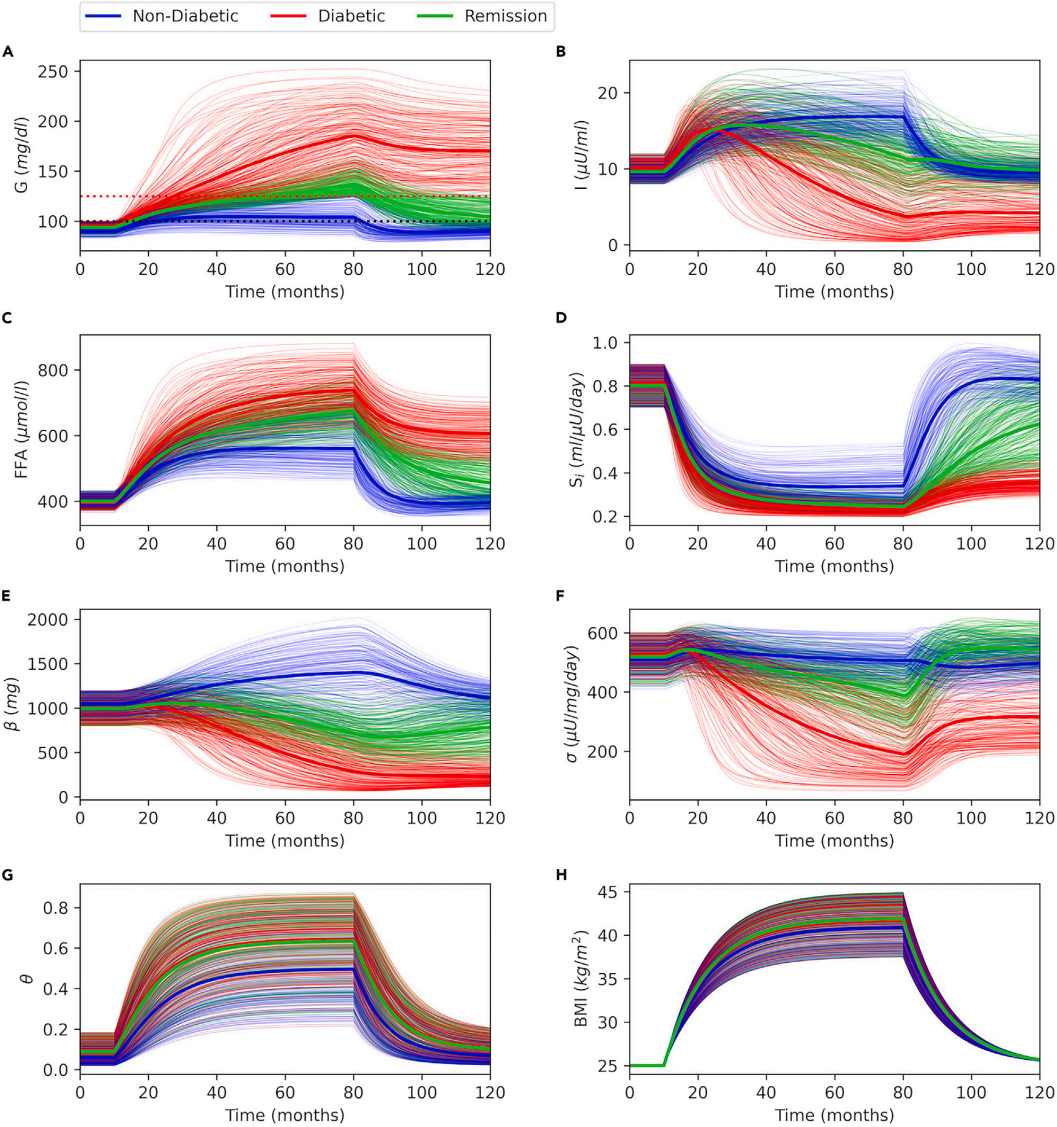
Trends in a virtual population generated through Monte Carlo sampling (A) Basal glucose with prediabetes (black dotted line, 100 *mg/dL*) and T2D cutoff (red dotted line, 125 *mg/dL*). (B) Plasma insulin, (C) plasma FFA, (D) insulin sensitivity, (E) β-cell mass, (F) β-cell function, (G) inflammation index, and (H) BMI time courses. At T = 10 months, daily energy intake (*DE*_*i*_) is increased from baseline by a randomly chosen value between 50% and 80% to simulate different weight gain scenarios. At T = 80 months, DE_i_ is set back to the baseline value to simulate a diet intervention for each individual. The virtual population is classified into three subgroups: non-diabetics (blue), diabetics (red), and remission (green). For each group, 200 traces were randomly selected for illustration purposes, where bold traces represent group averages.

In order to understand the progression of T2D and remission through different metabolic profiles, we investigate a population of virtually generated individuals by randomly sampling model parameters (see Figure S9 in supplemental information for details of randomly sampled model parameters). For each virtual individual, model simulations were generated for 120 months, where at T = 10 months, DE_i_ is increased from baseline by a randomly chosen value between 50% and 80% to simulate different weight gain scenarios. At T = 80 months, DE_i_ is set back to the baseline value to simulate a diet intervention. For each simulation, the first 10 months mark the baseline phase, the range between 10 and 80 months is the weight gain phase, and the time between 80 and 120 months is the intervention phase. Afterward, the virtual population is classified into three subgroups: non-diabetics, diabetics, and remission. Figure 6 shows 200 randomly selected traces from each group. The non-diabetic group comprises the individuals whose glucose levels do not rise to the T2D ranges (Figure 6A, red dotted line at 125 mg/dL) throughout the simulation time frame (Figure 6, blue); the diabetic group comprises individuals whose glucose levels rise to the T2D range and remain in this range for the rest of the simulation time frame (Figure 6, red); and the remission group comprises the individuals whose glucose levels rise to the T2D ranges but fall back after the intervention (Figure 6, green).

For the first 10 months, BMI is 25 kg/m^2^ for each simulation (Figure 6H), and other variables remain at their respective baselines. As the weight gain is initiated at T = 10 months, different patterns start to emerge, and groups begin to be stratified. For *G*, *FFA*, *S*_*i*_, *θ*, and *BMI*, overall trends are similar across groups. With weight gain, *G*, *FFA*, *θ*, and *BMI* are increased, and *S*_*i*_ is decreased monotonically until the intervention point, where they switch direction. However, *I*, *σ*, and *β* follow different trends for each group. All groups exhibit hyperinsulinemia at the early stages of the weight gain phase (Figure 6B), but only the non-diabetic group maintains hyperinsulinemia throughout the weight gain phase (Figure 6B, blue). For the remission group, decline in insulin starts later compared with the diabetic group, and it does not drop as much (Figure 6B, green versus red). After the intervention, insulin levels drop for the non-diabetic group due to the reduced glucose, *σ*, and *β*. However, for the diabetic and remission groups, insulin does not change much after the intervention. In the non-diabetic group, *σ* and *β* increase throughout the weight gain phase, and they decline to their baseline values after the intervention (Figures 6E and 6F, blue). Contrary to this, for the diabetic and remission groups, both *σ* and *β* are declined during the weight gain phase. Even though *σ* increases for both the diabetic and the remission groups after the intervention, *β* is only increased in the remission group (Figure 6E, green versus red).

**Figure 7:**
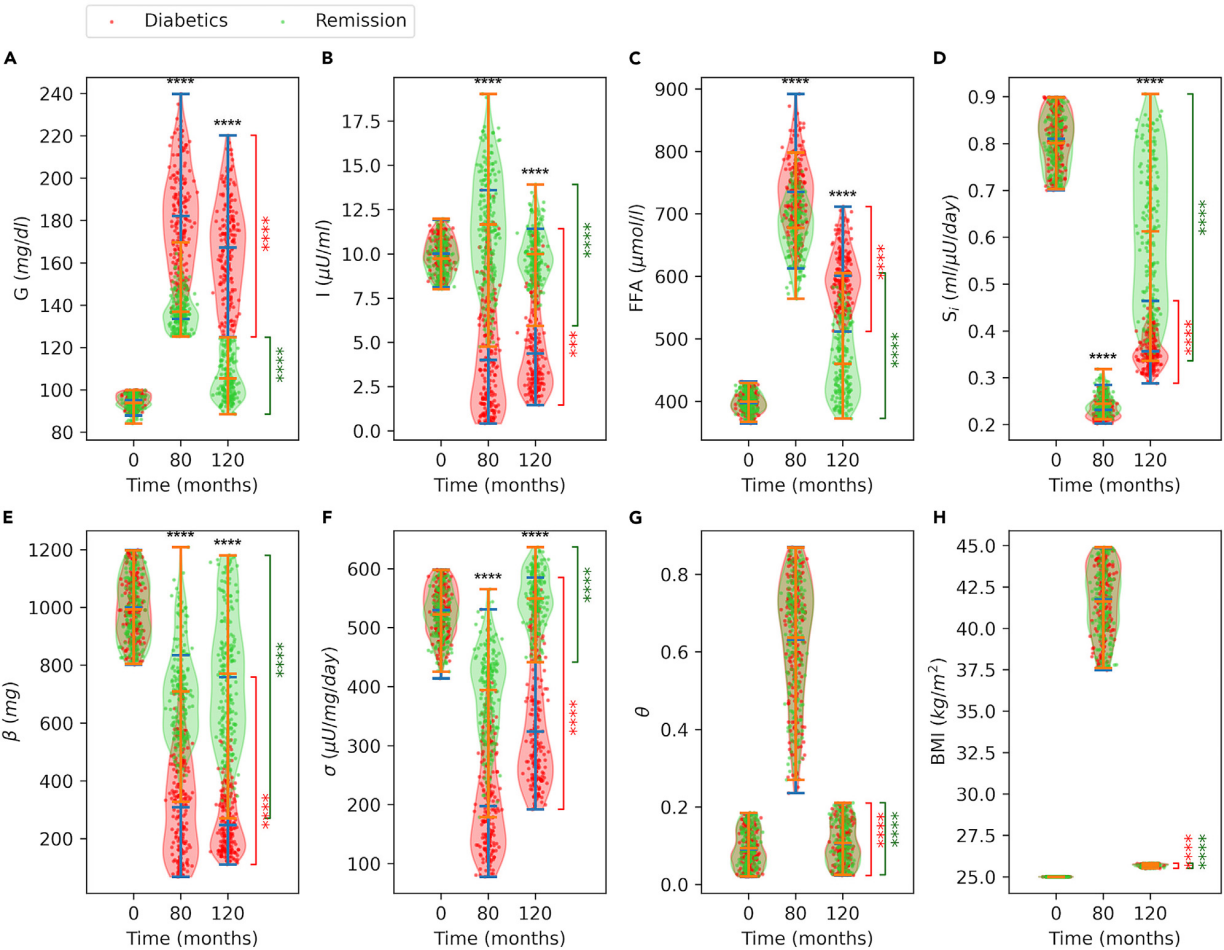
Distribution of diabetic and remission groups at different stages Violin plots of the model variables with individual data points at the beginning (T = 0), at the intervention time point (T = 80 months), and at the end of the simulations (T = 120 months) given in Figure 6. (A) Plasma glucose, (B) plasma insulin, (C) plasma FFA, (D) insulin sensitivity, (E) β-cell mass, (F) β-cell function, (G) inflammation index and (H) BMI. Bars show the minimum, median and maximum values of the distributions. Significance levels on the top indicate the difference between groups at corresponding time point, whereas the ones on the right compare the corresponding group means at T = 80 and T = 120 months. Significance levels: ∗p < 0.05, ∗∗p < 0.01, ∗∗∗p < 0.005, ∗∗∗∗p < 0.001.

Figure 6 shows that metabolic variables are distributed over relatively narrow ranges at the baseline, where the non-diabetic, diabetic, and remission groups can hardly be distinguished. However, as weight gain progresses over time, the distribution ranges become wider, and groups become evident. In Figure 7, we show the distribution of variables at the beginning (T = 0), at the intervention time (T = 80 months), and at the end of the simulations (T = 120 months) for diabetic and remission groups. At the baseline, diabetic and remission groups exhibit similar distributions over relatively narrow ranges, except for BMI, which is fixed to 25 kg/m^2^ for all simulations. However, at the intervention time (T = 80 months), two groups become apparent. At the intervention time, the remission group has higher insulin levels (Figure 7B) despite lower glucose (Figure 7A), which is a consequence of higher β-cell function and mass (Figures 7E and 7F). FFA levels are also lower for the remission group at the intervention (Figure 7C). On the other hand, both groups have very similar insulin sensitivity (Figure 7D), inflammation (Figures 7B and 7G), and BMI (Figure 7H) distributions at the intervention time.

At the end of the simulations, plasma glucose levels drop in both groups (Figure 7A), but for different reasons. In the remission group, the decline in glucose is a consequence of improved insulin sensitivity (Figure 7D), because insulin levels are actually reduced in this group after the intervention. On the other hand, in the diabetic group, decline in glucose levels following the intervention is a consequence of increased insulin levels (Figure 7B) in combination with slightly improved insulin sensitivity (Figure 7D). After the intervention, insulin follows different trends for each group. By the end of the simulation, plasma insulin drops in the remission group but it is increased in the diabetic group (Figure 7B). In the remission group, the decline in the insulin levels by the end of the simulations is a consequence of reduced glucose levels, because β-cell mass (Figure 7E) and β-cell function are actually increased (Figure 7F). However, in the diabetic group, insulin levels are slightly increased after the intervention, as a consequence of increased β-cell function (Figure 7F) despite the decline in β-cell mass (Figure 7E).

### The success of a weight management program for T2D remission depends on functional β-cell mass

**Figure 8:**
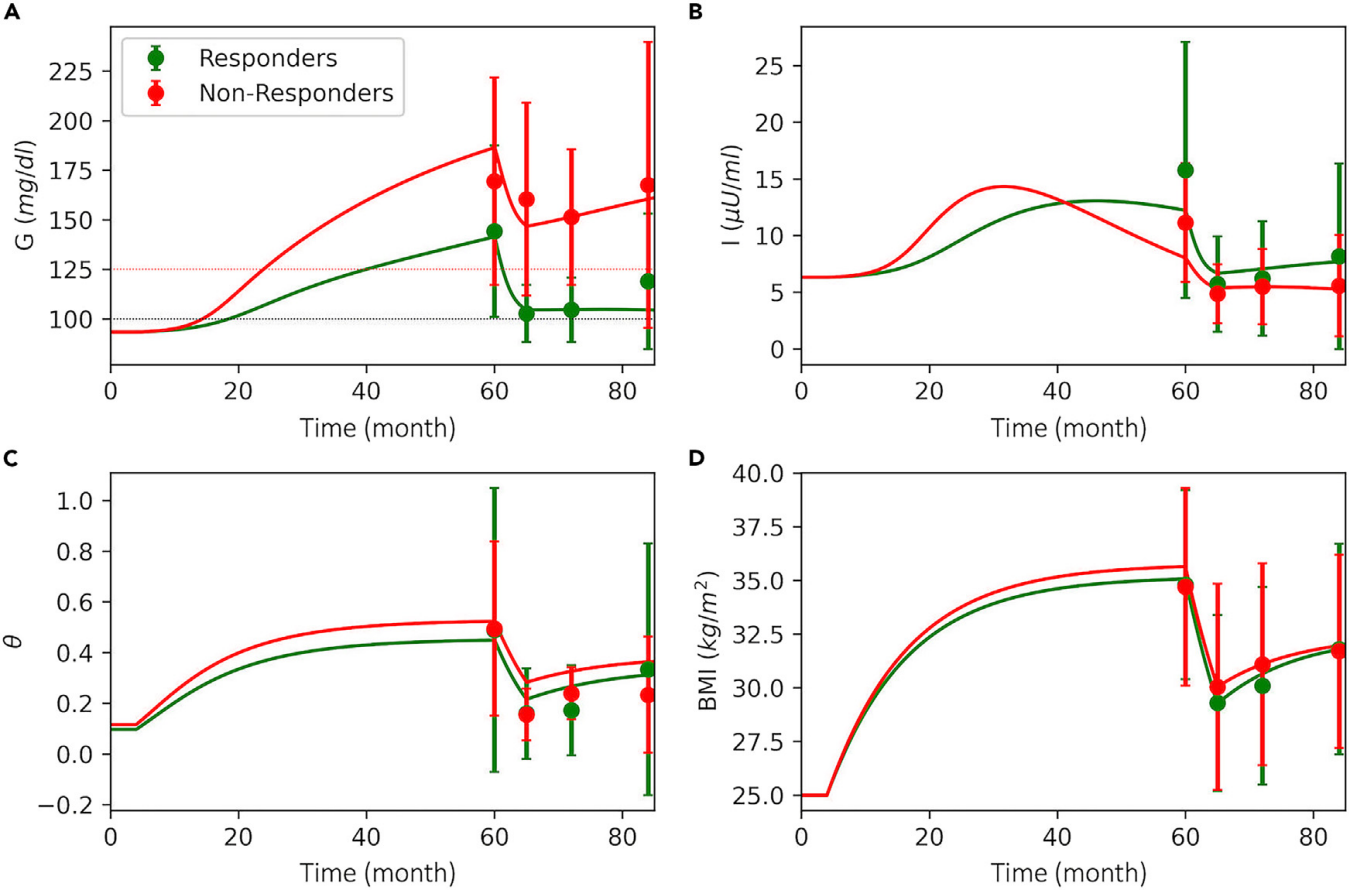
DIRECT study data against model simulations (A) Basal glucose with prediabetes (horizontal black dotted line, 100 *mg/dL*) and T2D cutoffs (red horizontal dotted line, 125 *mg/dL*). (B) Plasma insulin, (C) inflammation index, and (D) BMI time courses. The weight gain phase is initiated at T = 5 months by increasing the daily energy intake (*DE*_*i*_). At T = 60 months, high-intensity diet intervention phase is initiated. At T = 65 months, low-intensity weight maintenance phase is started. In each panel, filled circles with error bars show data adapted from (Mrabeh et al., 2020)^38^ as mean ± standard deviation, and solid curves show model simulation.

Diabetes remission clinical trial (DIRECT) study was designed to investigate the feasibility and effectiveness of a weight management program for T2D remission. The program comprised up to 5 months low calorie diet intervention followed by a low-intensity weight maintenance phase for 24 months (see STAR Methods for details). The participants who achieved remission during the program were classified as responders (Figure 8 filled green circles with error bars), whereas those who failed in remission were classified as non-responders (Figure 8, filled red circles with error bars). We use these data to calibrate model for responders (Figure 8, green solid curve) and non-responders (Figure 8, red solid curve). The model is able to accurately capture the post-intervention dynamics in each group, which allows us to investigate the differences between responders and non-responders.

**Figure 9:**
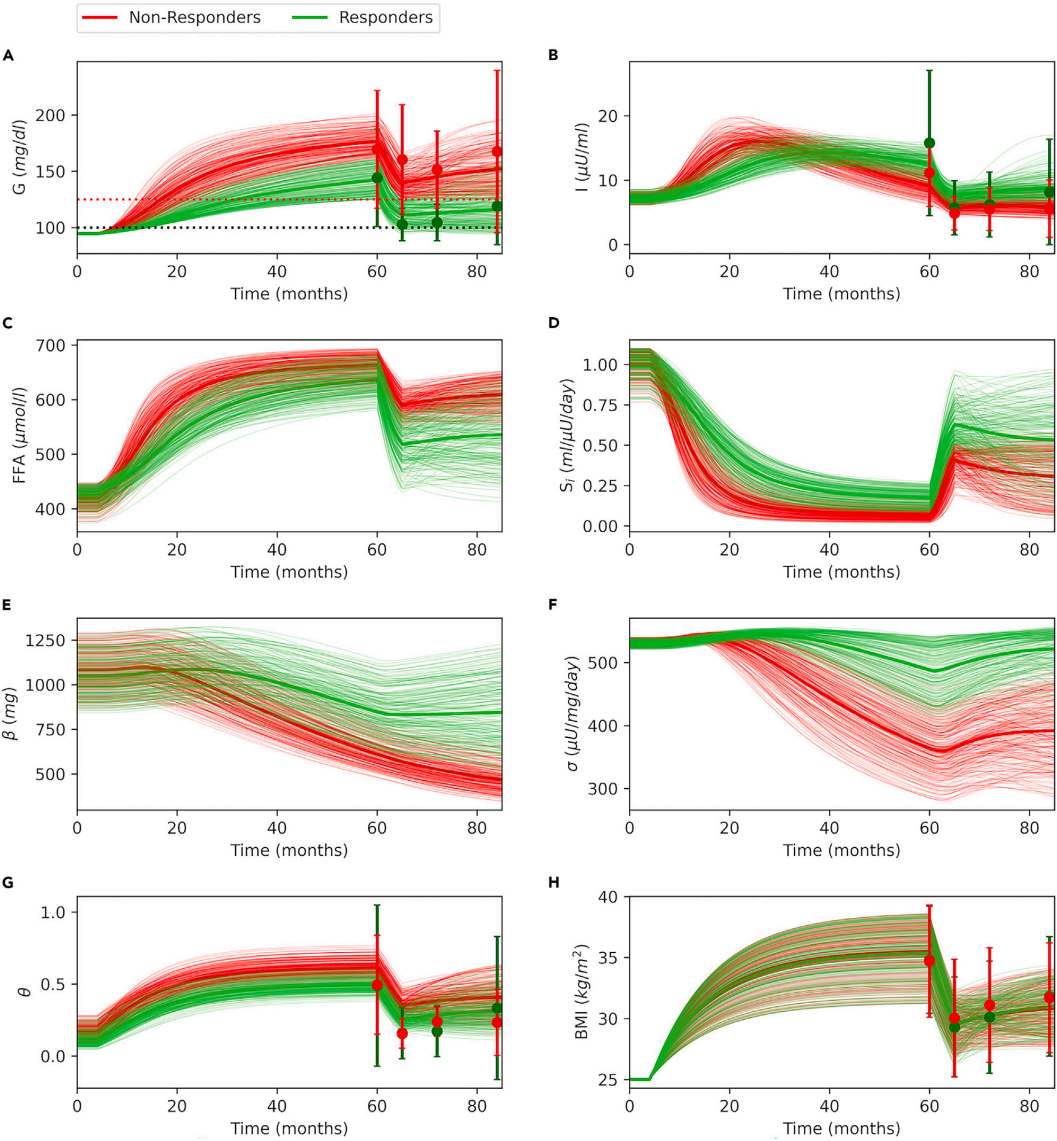
A randomly generated population that resembles DIRECT study cohort (A) Basal glucose with prediabetes (horizontal black dotted line, 100 *mg/dL*) and T2D cutoffs (red horizontal dotted line, 125 *mg/dL*). (B) Plasma insulin, (C) plasma FFA, (D) insulin sensitivity, (E) β-cell mass, (F) β-cell function, (G) inflammation index, and (H) BMI time courses. At T = 5 months, daily energy intake (*DE*_*i*_) is increased from baseline by a randomly chosen value between 125% and 155% of the baseline to simulate different weight gain scenarios. At T = 60 months, DE_i_ is set back to a value between 60% and 80% of the baseline to simulate a diet intervention for each individual. Finally, at T = 72 months, DE_i_ is increased to a value between 110% and 140% of the baseline to simulate the stepped food reintroduction phase as in DIRECT study. The population is classified as non-responders (red) and responders (green). For each group, 50 traces were randomly selected for illustration purposes. Bold traces represent group averages, whereas filled circles and error bars show data adapted from (Mrabeh et al., 2020)^38^ as mean ± standard deviation.

To investigate the progression dynamics of T2D and gain more insights into the metabolic differences between responders and non-responders, we generated a virtual population that resembles the DIRECT study group by randomly sampling calibrated model parameters and simulating the DIRECT study weight management program (Figure 9, see Table S5 and STAR Methods for details). Although, there are no data available for the pre-intervention phase of the study, the model allows us to make predictions regarding the progression history of T2D and compare both groups. Our results show that the overall BMI trends are similar for both groups (Figure 9H). However, responders have lower systemic inflammation (Figure 9G). Average T2D duration at the intervention is 27.2 ± 10.1 months for the responders (Figure 9A, green) and 42.3 ± 3.4 months for the non-responders. In both groups, insulin levels increase at the early stages of T2D onset (Figure 9B) following the hyperglycemia. In non-responders, insulin peaks to higher levels compared with responders (Figure 9B). However, only responders maintain high insulin levels until the intervention time point (Figure 9B, green), whereas, in non-responders, insulin levels start to decline at very early stages of the T2D onset (Figure 9B, red). The model also shows that both groups have similar FFA (Figure 9C) and *S*_*i*_ (Figure 9D) levels at the baseline (0–5 months), but responders have lower FFA (Figure 9C) and higher *S*_*i*_ (Figure 9D) during the weight gain phase (5–60 months) and after the intervention (60–85 months). A similar trend is evident for both groups for β-cell mass (Figure 9E) and β-cell function (Figure 9F), where both groups have similar baseline values, but responders maintain higher β-cell mass (Figure 9E, green) and β-cell function (Figure 9F, green) during the T2D progression phase (5–60 months). During the intervention phase (60–85 months), β-cell function improves in both groups (Figure 9F), but β-cell mass only increases in the responders (Figure 9E). This shows that one of the key differences between responders and non-responders is that the former group has a higher functional β-cell mass at the intervention, and it gradually improves following the weight loss.

## Discussion

In this study, we developed a physiology-based computational model that explicitly describes weight gain dynamics to investigate the long-term effects of obesity on the progression of T2D and remission through weight loss. In the past, several computational models have been proposed to investigate the progression of T2D.^29^^,^^30^^,^^31^^,^^32^^,^^33^^,^^34^^,^^35^^,^^36^^,^^37^^,^^39^ However, these models do not account for the dynamics of the weight gain and associated metabolic changes that take place over time. Recently, Yang and colleagues extended a diabetes progression model by introducing a hypothetical obesity-related impairment factor, which implicitly represents pathogenic effects of weight gain on glycemic control.^40^ By explicitly describing the weight gain dynamics, here, we decomposed the effects of obesity into FFA and inflammation components to investigate the impact of weight gain on different metabolic pathways. One of the major contributions of the current study is that it allows us to study the dynamics of the T2D progression with weight gain and remission through weight loss on the same timeline. This is not possible through experimental and observational studies due to the limitations that arise from the multifactorial and slow nature of T2D progression. Therefore, the current study reproduces experimental and observational data and provides deeper insights. Our results show that, in individuals with obesity, in addition to a decreased insulin sensitivity and reduced β-cell function, a substantial decline in β-cell mass is also necessary for the emergence of overt T2D (Figure 2). However, the model predicts that T2D remission does not necessarily require β-cell mass to be recovered (Figure 4E), where improved insulin sensitivity (Figure 4D) and increased β-cell function (Figure 4F) could be sufficient. Our results explain why remission through weight loss is only possible within a window of opportunity. That window depends on the progression history of T2D, the individual’s metabolic state, and level of calorie restriction (Figure 4). Our model predicts that the prevention of T2D is metabolically less challenging than the remission (Figure S7), and a faster weight gain is more detrimental to glycemic control compared with weight gain at a slower pace (Figure S8).

The current study not only reproduces experimental and observational data accurately but also provides new insights into the progression dynamics of T2D and remission through weight loss. Observational studies that focus on the weight loss and diabetes remission are limited to certain metabolic measurements after the intervention, which does not give information about the progression of T2D. In addition to the multifactorial, slow, and inconspicuous nature of diabetes,^27^^,^^28^ medical and/or ethical concerns also limit the collection of such comprehensive data. Because, certain measurements, such as β-cell mass, require invasive procedures, and hence such studies usually rely on postmortem tissue samples collected from donors.^41^^,^^42^^,^^43^ Furthermore, the design of the clinical studies that measure functional β-cell capacity^15^^,^^19^ does not allow to differentiate β-cell mass from β-cell function.^44^^,^^45^ The current study fills this gap by providing the opportunity to study the emergence dynamics of T2D with weight gain and remission through weight loss on the same timeline and in the same study group. Therefore, the model allows utilizing post-intervention data for making predictions about the progression history of T2D, which allows gaining deeper insights into the differences between individuals who achieve remission through weight loss and those who fail. To this end, we utilized previously published diabetes remission clinical trial (DIRECT) study data.^38^ For the DIRECT study group, our model predicts a longer T2D duration for non-responders compared with responders (Figure 9A, 27.2±10.1 versus 42.3 ± 3.4 months), which is consistent with the T2D diagnosis records published in the DIRECT study (32.4 ± 19.2 versus 45.6 ± 20.4 months). The model also shows that one of the key differences between responders and non-responders is that, during the emergence and progression of T2D, the former group is able to maintain hyperinsulinemia, whereas the latter is not (Figure 9B). The model also shows that, after the intervention, β-cell function increases in both groups (Figure 9F) but β-cell mass only increases in responders (Figure 9E). DIRECT study magnetic resonance imaging (MRI) data show a higher pancreas volume in non-diabetic controls compared with study participants with T2D at the baseline and a greater increase in pancreas volumes of the responders compared with non-responders after the intervention.^38^ Furthermore, DIRECT study MRI data also show irregularities in the pancreas morphology in the participants with T2D, which only normalizes in the responders after the intervention. Although lower pancreas volume and irregularity are not directly related to endocrine tissue mass or function, they are argued as potential risk factors or indicators for the development of T2D.^38^

In people with insulin resistance and obesity, insulin secretion is increased due to increased β-cell function^46^ and β-cell mass.^41^^,^^43^ Studies performed on endocrine tissue samples collected from the pancreases of postmortem donors show a significantly higher β-cell mass in normoglycemic individuals with obesity when compared with lean matches.^42^^,^^47^ Based on these findings and earlier modeling work,^29^^,^^35^^,^^36^ we hypothesize that during the early stages of obesity-driven insulin resistance, β-cell function and β-cell mass are increased, which induces hyperinsulinemia and keeps plasma glucose under control. Our population simulations show that β-cell mass can increase up to 2-fold with obesity (Figure 8E), which is consistent with earlier reports showing between 20% and 90% increase in β-cell mass in people with obesity.^41^^,^^43^ On the other hand, elevated glucose and lipid levels^5^^,^^48^ and systemic inflammation^6^^,^^49^ cause β-cell dysfunction and death in people with obesity and T2D.^7^^,^^50^ It has been suggested that pancreatic T2D takes place after fractional β-cell surface area declines by approximately 65%.^51^ Recently, it has been shown that deterioration in β-cell insulin secretion capacity, not insulin sensitivity, is a determinant of impaired fasting glucose that ultimately leads to T2D in people with obesity.^52^ Indeed, our results show that, in addition to insulin resistance, a substantial decline in β-cell mass is necessary for the emergence of T2D as suggested before.^53^ Our model predicts that more than 60% decline in β-cell mass would result in irreversible T2D (Figures 3B and 7J). The current study also confirms that the decline in functional β-cell mass starts long before the diagnosis of T2D.^17^^,^^44^ These findings imply that, in people with obesity, by the time T2D is diagnosed, functional β-cell mass may already be at critically low levels, which would make remission more challenging.

Observational studies suggest that people can remain in an insulin-resistant prediabetic state for 10 years on average before they progress to an overtly diabetic state.^54^^,^^55^ These studies emphasize that, with lifestyle modifications such as diet and exercise, prediabetes can easily be resolved with lasting remission.^56^ Our results show that plasma glucose levels can remain within the prediabetic range for years, only if body weight is maintained under a certain threshold (Figure 3, green). Furthermore, with an early weight loss intervention, prediabetes or even T2D can be fully resolved (Figure 4, blue). Interventions that take place earlier in time or at lower BMI levels have a higher chance to induce remission with sufficient calorie restriction (Figure 4, green). The model proposed by Ha and colleagues also shows that early interventions that improve insulin sensitivity can resolve prediabetes and result in lasting remission.^35^ Observational studies suggest that, after T2D onset, there is up to a 2.7 years of window of opportunity for remission through weight loss.^14^^,^^15^^,^^18^^,^^19^ Although some studies found no correlation between the success of remission and the T2D duration,^26^ it has been found that earlier interventions increase the chances for remission.^18^^,^^19^ Our results confirm the existence of such a window of opportunity, where we explain this phenomenon via the bistability and the thresholds emerging in the system dynamics (Figures 3 and 4). Earlier modeling studies also suggest the existence of thresholds that separate metabolic states.^29^^,^^35^^,^^37^ By explicitly modeling the weight gain dynamics, the current study defines these thresholds in relation to daily energy intake and BMI. Observational studies also suggest a more than 60% increase in functional β-cell capacity within the first 12 months of weight loss interventions, which does not change much afterward.^14^^,^^15^ Our model predicts that observed recovery in functional β-cell capacity in these studies is most likely due to increased β-cell function rather than β-cell mass, because β-cell mass does not change much within the first 12 months of intervention (Figure 4, green).

Genetic factors, lifestyle, other medical conditions, and psychological state may affect weight gain dynamics.^57^ For instance, chronic stress affects weight gain dynamics through behavioral and hormonal changes,^58^^,^^59^ whereas most antipsychotic drugs^60^ and antidepressants^61^ induce a fast weight gain. Our model predicts that it is more challenging to resolve T2D that emerges as a result of fast weight gain (Figure S8, magenta in supplemental information). Due to the nonlinear interactions between system components, fast weight gain puts a greater burden on the metabolism in a relatively short period of time. Hence, the metabolic system fails in sufficiently adapting to these changes (Figure S8J, magenta in supplemental information). Earlier modeling work also suggests that a faster deterioration in insulin sensitivity is more likely to result in T2D compared with a slow gradual decay.^29^^,^^35^ Although, these models do not include weight gain dynamics as the underlying cause of the insulin resistance, they show the importance of the system’s dynamics. This result indicates that people with similar BMI and genetic profiles might have very different metabolic states simply because of their weight gain history. This introduces another level of complexity to the etiology of obesity-driven T2D.

Although we primarily focused on the development of insulin resistance and T2D with obesity, weight gain does not result in T2D in every individual. Indeed, a great proportion of individuals with obesity are metabolically healthy and have normal glycemic control.^62^^,^^63^^,^^64^^,^^65^ Our population simulations confirm that individuals with obesity can have different glycemic control ranging from normoglycemic to diabetic levels, even with a similar BMI (Figure 6H). Earlier reports show that metabolically healthier and insulin sensitive individuals with obesity have lower levels of pro-inflammatory mediators and lower plasma FFA levels.^62^^,^^66^^,^^67^ Consistent with this, our results show that inflammation has a significant impact on the system dynamics, where it accelerates the progression of T2D and shrinks the window of opportunity for remission (Figure 5). On the other hand, the interaction between FFA, insulin secretion, and insulin sensitivity is more complex and constitutes feedback loops. Therefore, higher FFA levels seen in individuals with obesity and T2D are both a cause and a consequence of the T2D (Figures 6C and S10C in supplemental information).

In conclusion, in individuals with obesity, T2D emerges with reduced insulin sensitivity followed by impaired β-cell function and reduced β-cell mass. Our model substantiates the hypothesis that the prevention of T2D is metabolically less challenging than remission. The model is able to reproduce observational data collected through a diabetes remission clinical trial and provide deeper insights into the progression dynamics of T2D and the differences between the individuals who achieve remission through weight loss and those who do not. Our results show that remission is only possible within a window of opportunity with sufficient calorie restriction that depends on T2D duration and the remaining functional β-cell mass. Furthermore, weight gain history and the level of inflammation are important factors in the etiology of T2D, which also determine the success of remission after weight loss.

### Limitations of the study

In this study, we focused on the long-term slow dynamics of the obesity-driven changes in metabolic variables at their basal fasting states. Although we were able to show the way the basal state of the metabolic system changes in response to weight gain, we did not investigate the postprandial state dynamics. Although individual’s glycemic control or diabetes status are usually determined by the fasting state measurements, such as fasting plasma glucose^68^ or homeostatic model assessment insulin resistance index (HOMA-IR),^69^ postprandial measurements, such as oral glucose tolerance tests, can give more accurate information about insulin resistance and T2D status.^70^ Furthermore, postprandial metabolic responses are shown to be better correlates for cardiometabolic risk assessment.^71^ In the current model, the fast variables of the system (glucose, insulin, and FFA) operate in a quasi-steady state, which slowly change over time following the dynamics of the slow variables. This approach allowed us to simulate long-term system dynamics over years with a relatively low computational cost, and it also made dynamical analyses and interpretation of the results easier. However, in the postprandial state, fast system dynamics operate on the order of minutes. Therefore, incorporating postprandial state dynamics into the model significantly increases the computational cost, which makes it practically impossible to investigate the long-term slow dynamics for a large virtual population. Nevertheless, in the future, including postprandial state dynamics into the model can provide new insights into the progression of T2D. Furthermore, incorporating a physiologically relevant gastrointestinal module into the model could allow to investigate the impact of different diet regimens, such as a lipid-rich diet, and daily eating habits (e.g., frequently consumed small amounts versus three regular meals a day) on the progression of T2D. This could also allow us to compare conventional weight loss methods with the non-conventional procedures, such as bariatric surgery. Studies suggest that caloric restriction and bariatric surgery may result in improvements in glycemic control long before a significant weight loss takes place,^72^ indicating the weight-loss-independent benefits of these interventions. These early improvements are thought to be associated with negative energy balance following caloric restriction^73^^,^^74^ and increased incretin levels.^75^^,^^76^ In a recent study, we showed that GLP-1 levels are indeed significantly increased in Roux-en-Y gastric surgery patients at one-year follow-up along with improved insulin responsiveness in lipid and carbohydrate pathways.^77^ Although neither the current version of the model allows studying these weight-loss-independent early improvements associated with postprandial responses nor these early measurements are available in DIRECT study, these questions can be pursued by an extended version of the model that includes fast postprandial state dynamics.

## STAR★Methods

### Key resources table

**Table:** 

REAGENT or RESOURCE	SOURCE	IDENTIFIER
**Deposited data**		

	Mrabeh et al., 2020^38^	https://github.com/vehpi/obesity_T2D.git

**Software and algorithms**		

Python 3.10.6	Python Software Foundation	https://www.python.org
XPPAUT 8.1	XPP/AUTO, Dynamical Systems Analysis and Integration Toolbox,^78^	https://sites.pitt.edu/∼phase/bard/bardware/xpp/xpp.html

**Other**		

Model code	This study	https://github.com/vehpi/obesity_T2D.git

### Resource availability

#### Lead contact

Further information requests should be directed to and will be fulfilled by the lead contact Vehpi Yildirim (v.yildirim@amsterdamumc.nl).

#### Materials availability

No new materials or reagents were generated during this study.

#### Data and code availability

• This paper analyzes existing, publicly available open access data. These accession numbers for the datasets are listed in the key resources table, and details are discussed in the text.
• The source codes used to perform the analysis are publicly available as of the date of publication, and they are listed in the key resources table.
• Any additional information required to reanalyze the data reported in this paper is available from the lead contact upon request.

### Experimental model and study participant details

Diabetes remission clinical trial (DIRECT) study is a cluster randomized trial designed to investigate the feasibility and effectiveness of a weight management program for T2D remission.^79^ We use a subset of the previously published summary data for a cohort of adult DIRECT study participants, who had T2D at the baseline, and participated in a magnetic resonance imaging (MRI) procedure.^38^ Participants were aged between 20–65 years, who had been diagnosed with T2D within the past 6 years, with a BMI of 27–45 kg/m^2^. In short, participants were randomly assigned to a weight management intervention or routine diabetes control groups. The intervention group received a low-calorie liquid formula diet [825–853 kcal/day] for 3 to 5 months, which was followed by stepped food reintroduction and a low-intensity weight maintenance phase for up to 24 months. HbA1c, fasting glucose, fasting insulin, BMI, C-reactive protein levels and pancreas MRI data were collected at the baseline and during 5-, 12- and 24-month follow-ups. Intervention group was further categorized into responders (with HbA1c <6.5% [48 mmol/mol] and fasting blood glucose <7.0 mmol/L, without use of any antidiabetic drugs) and non-responders, who failed in remission after the intervention. For details of the dataset see Table S4 in supplemental information.

### Method details

#### Weight gain model

The weight gain model was adapted from (Crielaard et al., 2020),^80^ where the rate of change of body weight (*W*) is defined as a function of daily energy intake as follows;

(Equation 1) $$\frac{dW}{dt}=k_{w}{DE}_{i}-{DE}_{e}W$$

Where, *k*_*w*_ is the amount of weight gain per calorie, *DE*_*i*_ is the total daily energy intake in calories and *DE*_*e*_ is the rate of weight loss due to energy expenditure. BMI is calculated as a function of *W* as follows;

(Equation 2) $$BMI=\frac{W}{h^{2}}$$

Where *h* is height in meters.

#### Insulin-glucose (*I-G*) model

The insulin-glucose model was adapted from previously published mathematical models,^29^^,^^35^ which describe the dynamics of fasting plasma glucose (*G*) and insulin (*I*) concentrations over years as T2D progresses. In the model, plasma glucose is captured as a single compartment, which is sufficient to describe the glucose kinetics in the fasting state as suggested before.^29^^,^^81^^,^^82^ The rate of change of basal plasma glucose concentration is defined as

(Equation 3) $$\frac{dG}{dt}=HGP-\left(E_{G0}+S_{i}I\right)G$$

where, HGP is the hepatic glucose production rate, *E*_*G0*_ is the glucose effectiveness that represents insulin independent glucose uptake rate, *S*_*i*_ is insulin sensitivity and *I* is the plasma insulin concentration. HGP is adapted from (Ha and Sherman, 2020),^37^ where the HGP was defined as a function of *I* and *S*_*i*_ acting on the order of minutes to describe the fast dynamic responses to meals as well as intravenous or oral glucose tolerance tests. We adapted a simpler version of the HGP model to describe hepatic glucose production at fasting state as follows;

(Equation 4) $$HGP={HGP}_{b}+{HEPA}_{\max}\frac{\alpha_{HGP}}{\alpha_{HGP}+{HEPA}_{Si}I}$$

Here, HGP is defined as a decreasing sigmoid function of the product of hepatic insulin sensitivity (*HEPA*_*si*_) and plasma insulin level to account for the insulin mediated suppression of the hepatic glucose production. We set *HEPA*_*si*_ to equilibrium with peripheral insulin sensitivity (*S*_*i*_), since a significant correlation between peripheral and hepatic insulin resistance exists in individuals with diabetes.^83^ The rate of change of plasma insulin is defined by the following differential equation;

(Equation 5) $$\frac{dI}{dt}=\beta \frac{ISR}{V}-kI$$

Where *k* is insulin excursion rate, *V* is volume of distribution or plasma volume, and *ISR* is the rate of insulin production/secretion per unit β-cell mass (*β*), and it is defined by the following sigmoid function adapted from (Ha et al., 2016);^35^

(Equation 6) $$ISR=\sigma \frac{M^{K_{ISR}}}{M^{K_{ISR}}+{\alpha_{ISR}}^{K_{ISR}}}$$

Where *σ* is the maximal β-cell insulin production/secretion capacity (β-cell function) and *M* is the β-cell metabolic rate, which is adapted from (Ha et al., 2016)^35^ as a sigmoid function of *G,* as follows;

(Equation 7) $$M=\frac{G^{K_{M}}}{G^{K_{M}}+{\alpha_{M}}^{K_{M}}}$$

In Equations 6 and 7, parameters *α*_*ISR*_ and *α*_*M*_ determine the positions of the response curves vs. *M* and *G*, respectively, whereas *K*_*ISR*_ and *K*_*M*_ determine the steepness of the curves.

#### Plasma free fatty acids (*FFA*) model

The plasma free fatty acids (FFA) model was adapted from (Periwal et al., 2008),^84^ where the rate of change of plasma FFA is given by the difference between plasma FFA rate of appearance (*FFA*_*Ra*_) and disappearance (*FFA*_*Rd*_), whereas insulin mediated suppression of adipose tissue lipolysis is captured by defining *FFA*_*Ra*_ as a decreasing function of the insulin action as follows;

(Equation 8) $$\frac{dFFA}{dt}=\overset{{FFA}_{Ra}}{\overset{︷}{{FFA}_{Ra,\max}\frac{1}{1+{\left(\frac{S_{i}I}{K_{siF}}\right)}^{\alpha_{SiF}}}}}-\overset{{FFA}_{Rd}}{\overset{︷}{c_{f}\;W\;FFA}}$$

Here, *FFA*_*Ra*_ is defined as a decreasing function of the product of plasma insulin and insulin sensitivity, which is derived from the steady state approximation for the insulin action variable in (Periwal et al., 2008).^84^ We use a steady state approximation since we are interested in fasting FFA dynamics over the time course of years in relation to insulin sensitivity and insulin levels. *FFA*_*Ra,max*_ is the maximal rate of FFA production through adipose tissue lipolysis at zero insulin. *K*_*siF*_ is the half maximal response parameter and *α*_*SiF*_ is the Hill coefficient that determines the steepness of the sigmoid response curve. *FFA*_*rd*_ is defined as a function of body weight (*W*), where *c*_*f*_ is the FFA turnover rate per kg body weight.

To account for the impact of obesity and increased adipose tissue mass on the FFA kinetics, we extended this model by including dependence of plasma *FFA*_*Ra*_ on fat mass (*FM*). *FM* is calculated as the product of body weight (*W*) and the Fat Mass Ratio (*FMR*). Mittendorfer et al. show a linear relationship between *FFA*_*Ra*_ and *FM* in glucose tolerant individuals.^85^ Using the regression data extracted from Figure 1A of (Mittendorfer et al., 2009)^85^ by using GRABIT^86^ software package for MATLAB MathWorks, R2019b, we define *FFA*_*Ra,max*_ as follows;

(Equation 9) $${FFA}_{Ra,\max}=\left[F_{Ra0}+F_{Ra1}FM\right]$$

In Equation 9, maximal lipolysis rate is given as a linear function of *FM*, where *F*_*Ra0*_ and *F*_*Ra1*_ are the parameters that determine the linear relationship. *FMR* is calculated from the body mass index (BMI) using the formulation proposed before.^87^

#### Modeling systemic inflammation (*θ*) dynamics

Obesity is recognized as an inflammatory state with elevated levels of pro-inflammatory mediators such as tumor necrosis factor-alpha (TNF-α), interleukin-1-beta (IL-1-β), IL-6, C-reactive protein (CRP), leptin and reduced levels of adiponectin.^88^ Studies show a close correlation between these pro-inflammatory mediators and BMI.^89^^,^^90^^,^^91^^,^^92^ Rather than focusing on the relationship between each pro-inflammatory biomarker and metabolic health separately, the use of systemic inflammation scores has been proposed.^93^ These inflammation scores are able to successfully capture the association between systemic inflammation and metabolic abnormalities; such as hypertension,^94^ insulin resistance^8^ and T2D.^95^ Here we propose a similar approach and introduce a unitless systemic inflammation index (*θ*) as a dynamic variable that changes over time with BMI as follows;

(Equation 10) $$\frac{d\theta }{dt}=\frac{\theta_{\infty }-\theta }{\tau_{\theta }}$$

Where, *θ*_*∞*_ is the steady state function, and *θ* dynamically approaches this value with a rate proportional to the time constant *τ*_*θ*_. *θ*_*∞*_ is defined as an increasing sigmoid function of BMI in the following form;

(Equation 11) $$\theta_{\infty }=\frac{{BMI}^{n_{\theta }}}{{BMI}^{n_{\theta }}+K_{\theta }^{n_{\theta }}}$$

Here, *θ*_*∞*_ is an increasing sigmoid function of BMI that changes between [0, 1], where *n*_*θ*_ determines the steepness of the response and *K*_*θ*_ is the response parameter that defines BMI value for half maximal response.

#### Modeling insulin sensitivity (*S*_*i*_) dynamics

In order to understand the impact of the decline in insulin sensitivity on the progression of T2D, we adapt a model for the dynamics of the insulin sensitivity (*S*_*i*_) from a model proposed before.^29^^,^^35^ In the model, the rate of change of *S*_*i*_ is given by following differential equation;

(Equation 12) $$\frac{dS_{i}}{dt}=\frac{S_{i,\infty }-S_{i}}{\tau_{Si}}$$

In earlier models, *S*_*i,∞*_ is introduced as a control parameter that defines a steady state value, and *S*_*i*_ converges to this value over time with a rate proportional to the time constant *τ*_*Si*_.^29^^,^^35^ In obesity, an increased level of pro-inflammatory mediators is known to play a causative role in the development of insulin resistance.^6^^,^^9^ Moreover, it was shown that increasing plasma FFA levels by lipid injections lead to a significant decline in peripheral insulin sensitivity in humans within a few hours.^96^^,^^97^^,^^98^ In order to account for the effect of obesity-driven adiposity and inflammation on the dynamics of insulin sensitivity, we define *S*_*i,∞*_ as a function of plasma FFA and *θ* in the following form;

(Equation 13) $$S_{i,\infty }\left(FFA,\theta \right)=S_{i,b}\left[1-M_{FFA}\frac{{FFA}^{n_{Si}}}{{FFA}^{n_{Si}}+K_{Si,FFA}^{n_{Si}}}\right]\left[\frac{K_{Si,\theta }^{n_{\theta }}}{K_{Si,\theta }^{n_{\theta }}+\theta^{n_{\theta }}}\right]$$

The details of the way *S*_*i,∞*_ is related to *FFA* and *θ* are provided in the supplemental information (Figure S1).

#### Modeling β-cell mass (*β*) dynamics

The β-cell mass model is adapted from (Ha et al., 2016),^35^ which defines the rate of change of β-cell mass (*β*) as a balance between β-cell replication and β-cell loss. In this model, the β-cell replication rate is defined as an increasing sigmoid function of insulin secretion rate (*ISR*) and the β-cell apoptosis rate is defined as an increasing sigmoid function of β-cell metabolic rate (*M*). We adapt this model by introducing pairwise interaction between β-cells. This approach is physiologically more accurate, since β-cell mass cannot grow indefinitely due to limited resources (nutrition, oxygen, space, etc.). De Gaetano et al. used a similar approach before by assuming logistic growth with a fixed carrying capacity in their model.^36^ Here, we explicitly define a pairwise interaction term between β-cells, which is the basis for any logistic growth model. Using a logistic growth model results in a saddle-node bifurcation in system’s dynamics, which leads to a physiologically more sensible interpretation of dynamics compared to the trans-critical bifurcation shown in model by Topp and colleagues.^29^ We also introduce a term for neogenesis that represents differentiation of other endocrine cell types and duct cells into β-cells.^99^ With the current modification, the differential equation for the rate of change of β-cell mass (*β*) reads as follows;

(Equation 14) $$\frac{d\beta }{dt}=\frac{P_{ng}+P\left(ISR\right)\beta -A\left(M\right)\beta -s\beta^{2}}{\tau_{\beta }}$$

Where *P*_*ng*_ is the rate of β-cells neogenesis, *P(ISR)* and *A(M)* are the *ISR* dependent replication rate and *M* dependent apoptosis rate adapted from (Ha et al., 2016),^35^ respectively. *s* is the pairwise interaction term between β-cells and *τ*_*β*_ is the time constant that scales the dynamics.

(Equation 15) $$P\left(ISR\right)=P_{\max}\frac{{ISR}^{n_{P}}}{{ISR}^{n_{P}}+\alpha_{P}^{n_{P}}}$$

*P(ISR)* is defined as an increased sigmoid function of *ISR*, where *P*_*max*_ is the maximal replication rate, *α*_*p*_ is the ISR that generates half maximal response, and *n*_*p*_ determines the steepness of the response.

(Equation 16) $$A\left(M\right)={A_{b}+A}_{\max}\frac{M^{n_{A}}}{M^{n_{A}}+\alpha_{A}^{n_{A}}}$$

*A(M)* is defined as an increasing sigmoid function of *M*, where *A*_*b*_ is the constant apoptosis rate that is independent of *M*, *A*_*max*_ is the maximal *M* dependent replication rate, *α*_*A*_ is the *M* that generates half maximal response, and *n*_*A*_ determines the steepness of the response.

#### Modeling dynamics of β-cell function (*σ*)

In non-insulin resistant individuals with obesity, β-cell function and glucose stimulated insulin secretion (GSIS) is increased,^46^ whereas in individuals with obesity and insulin resistance or T2D, β-cell function is declined.^52^ This implies a nonmonotonic response β-cell function to weight gain. In obesity-driven T2D, the decline in β-cell function results from cytotoxic effects of hyperglycemia, increased lipid levels^48^ and inflammation.^6^^,^^49^ The toxic effects of glucose and lipids on β-cell metabolism are referred to as glucotoxicity and lipotoxicity, respectively. It is suggested that the toxic effects of lipids on β-cell function only become evident under hyperglycemic conditions.^100^^,^^101^ This phenomenon is called glucolipotoxicity, which signifies a synergistic detrimental effect of lipids and glucose on β-cell function.^102^ To describe the nonmonotonic behavior of β-cell function and its effect on the dynamics of T2D progression over the years, we adapted the mathematical formulation used in (Ha et al., 2016)^35^ to describe β-cell function (*σ*) dynamics by the following differential equation;

(Equation 17) $$\frac{d\sigma }{dt}=\frac{\sigma_{\infty }\left(G\right)-\sigma }{\tau_{\sigma }}$$

Where *σ*_*∞*_ defines an asymptotic value and *σ* approaches this value dynamically with a rate proportional to the time constant τ_σ_. In Ha et al., *σ*_*∞*_ is defined as a non-monotonic function that only depends on *ISR* and *M*, which are essentially functions of *G*.^35^ This way, they show that during the early stages of IR induced hyperglycemia, *σ*_*∞*_ is increased in response to increased *ISR,* and at later stages of IR or T2D, *σ*_*∞*_ is decreased in response to *M*. Since their model does not include FFA and inflammation dynamics, they omit the detrimental effects of these variables on *σ*. Based on the literature discussed above, we propose an alternative formulation for *σ*_*∞*_, where it depends on *G*, *FFA* and *θ* as follows;

(Equation 18) $$\sigma_{\infty }\left(G,FFA,\theta \right)=\sigma_{b}\left[\sigma_{gu}-\sigma_{gd}\sigma_{FFA}-\sigma_{\theta }\right]$$

Where *σ*_*b*_ is the baseline value for *σ*_*∞*_, and the term in brackets is set to 1 for the healthy baseline state. *σ*_*gu*_ is an increasing function of G and represents the hyperglycemia driven increase in *σ* during the early stages of IR. *σ*_*gd*_ represents the cytotoxic effects of glucose and *σ*_*FFA*_ represents cytotoxic effects of FFA on β-cell function, and their product defines glucolipotoxicity. Finally, *σ*_*θ*_ is an increasing sigmoid function of *θ* and accounts for the detrimental effect of inflammation on *σ*. The dependence of *σ*_*∞*_ on *G*, *FFA* and *θ* along with the details of *σ*_*gu*_, *σ*_*gd*_, *σ*_*FFA*_ and *σ*_*θ*_ are provided in the supplemental information (Table S1; Figure S2).

#### Diabetes progression index (*DPI*)

In the model, T2D emerges with insulin resistance and reduced insulin secretion due to the decline in β-cell function and mass. Therefore, we introduce the T2D progression index (*DPI*) as the product of the *S*_*i*_, *σ* and *β* normalized over their baseline values of *S*_*i,0*_, *σ*_*0*_ and *β*_*0*_, respectively (Table S2).

(Equation 19) $$DPI=1-\frac{S_{i}\sigma \beta }{S_{i,0}\sigma_{0}\beta_{0}}$$

This way, DPI is 0 at the baseline, and it increases over time with respect to the decline in *S*_*i*_, *σ* and *β*. The DPI is analogues to the disposition index, which gives information about the capacity of insulin secretion from β-cells in response to glucose (β-cell function) and the ability of insulin mediated glucose disposal (insulin sensitivity).^103^

#### Definition of metabolic states

In this study, we assume that a plasma glucose level between 100-125 mg/dl indicates prediabetes, whereas a plasma glucose level above 125 mg/dl indicates T2D, in accordance with American Diabetes Association criteria.^68^ We also assume that a BMI between 25-30 kg/m^2^ indicates overweight, whereas a BMI above 30 kg/m^2^ indicates obesity, in accordance with the WHO criteria for white, Hispanic and black populations, where BMI cutoffs might be different for other populations.^104^ In this paper, the healthy state or the baseline state is defined by a BMI of 25 kg/m^2^ and a plasma glucose level below the prediabetes cutoff (100 mg/dl).

### Quantification and statistical analysis

Data is presented as mean ± standard deviation. The statistical significance is tested using paired sample t-test, where either baseline and the intervention arms are compared, or the responders are compared to the non-responders. The model parameters ($\vec{p}$) are estimated using the method of maximum likelihood estimation (MLE). For normally distributed data with 0 mean, this corresponds to minimizing the following cost function ($J\left(\vec{p}\right)$) that is defined as the weighted sum of squared differences between model simulation and the data;

(Equation 20) $$J\left(\vec{p}\right)=\sum_{j=1}^{m}\sum_{k=1}^{T}\frac{{\left(y_{D,j}\left(t_{k}\right)-y_{j}\left(\vec{p},t_{k}\right)\right)}^{2}}{\sigma_{D,j,k}}$$

Where *y*_*D,j*_*(t*_*k*_*)* is the data for the *j*^*th*^ observation measured at the *k*^*th*^ time point and *σ*_*D,j,j*_ is the measurement error associated with this data point. In the estimation process, multi parameter searches were initiated from randomly selected points within the search space, and the parameter search that was resulted in minimum error was selected as the optimal parameter set, ${\vec{\theta }}^{*}$. For parameter standard deviations, asymptotic confidence intervals were calculated by using the covariance matrix *C=F*^*-1*^, where *F* is the Fisher Information matrix given by the negative hessian of the log-likelihood function calculated at the optimum point. We approximated the hessian of the log-likelihood by the first order model sensitivities given by the Jacobian matrix that is calculated during the parameter estimation, *H=J*^*T*^*J*.^105^ The parameter confidence interval for the *i*^*th*^ parameter is given by;

(Equation 21) $$\theta_{i}^{\pm }=\theta_{i}\pm t_{\alpha ,df}\sqrt{C\left(i,i\right)}$$

where, $t_{\alpha ,df}$ is the α quantile of the t-distribution with *df* degrees of freedom. Estimated model parameters and calculated confidence intervals for the population data are provided in Table S5.

#### Model calibration and computations

Model parameters were either directly obtained from literature, where available modeling and/or experimental human data was available, or submodules of the model were fit to the relevant human data available in the literature. The parameter values and their physiological explanations are provided in the supplemental information (Table S3), along with computer codes. For the parameter estimation non-linear least squares optimization function (*least_squares*) from python scientific computation toolbox is used (*scipy.optimize 1.7.3*).^106^ The sources of the data and parameter values are discussed in the text and also provided in the parameter table in the supplemental information (Tables S3 and S5). The system of differential equations was numerically solved in the Python programming environment using the *LSODA* algorithm, which is freely available within the integration module of the scientific computation package for Python (*scipy.integrate 1.7.3*).^106^ The numerical continuation analysis was carried out on the XPP/AUTO dynamical systems analysis and integration toolbox,^78^ and obtained data files were exported to Python to generate bifurcation diagrams. All model codes are available at https://github.com/vehpi/obesity_T2D.git, under MIT license.
