# Supplementary material for: A data-driven computational model for obesity-driven diabetes onset and remission through weight loss
Source: iScience. 2023 Oct 23;26(11):108324. doi: 10.1016/j.isci.2023.108324 (PMC10665812; doi:10.1016/j.isci.2023.108324)
Supplement: Document S1. Figures S1–S10 and Tables S1–S5 [file mmc1.pdf]

iScience, Volume *26*

Supplemental information

A data-driven computational model for obesity-driven diabetes onset and remission through weight loss

Vehpi Yildirim, Vivek M. Sheraton, Ruud Brands, Loes Crielaard, Rick Quax, Natal A.W. van Riel, Karien Stronks, Mary Nicolaou, and Peter M.A. Sloot

SUPPLEMENTAL INFORMATION

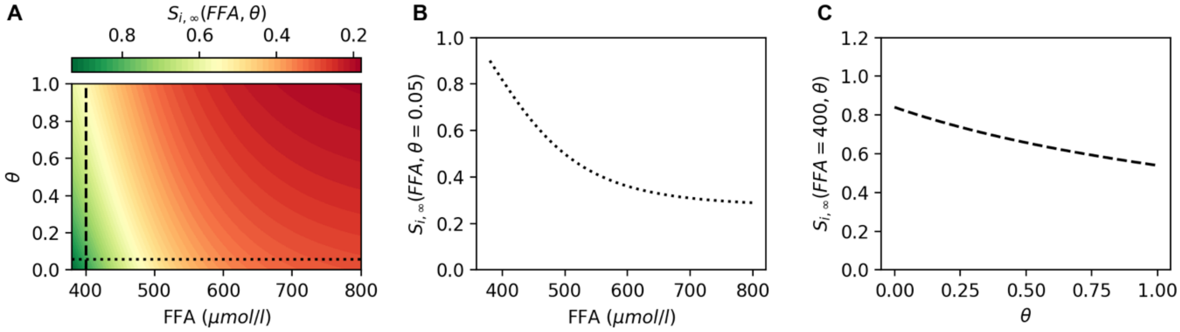

Figure S1: Association between $S_{i,\infty}$ and FFA and θ, related to STAR Methods. A) $S_{i,\infty}(FFA,\theta)$ surface (Eq. 13) is color mapped onto the $FFA x \theta$. B) The cross section of $S_{i,\infty}(FFA,\theta)$ surface at the baseline value of $\theta=0.05$ (Panel A, horizontal dotted line). C) The cross section of $S_{i,\infty}(FFA,\theta)$ surface at the baseline value of $FFA=400$ *μmol/l* (Panel A, vertical dashed line). At the baseline, $S_{i,\infty}$ is set 0.8 ml/μU/day in accordance with [2] (Panel A). The cross-sections of the $S_{i,\infty}$ surface for the baseline values of $\theta=0.05$ (Panel A, dotted-line and panel B) and $FFA=400$ *μmol/l* (Panel A, dashed-line and panel C) show the trend of decline in $S_{i,\infty}$ with respect to each variable. Boden et al. show that elevating plasma FFA levels to 750 *μmol/l* by lipid injection reduces insulin mediated glucose uptake by 55% within 6 hours in healthy subjects [3]. We use this data and represent FFA mediated decline in S_i (panel A, dotted line and panel B). We also use data extracted from Figure 1A in Recasens et al., 2005 [4] by using GRABIT [5] software package for MATLAB MathWorks, R2019b to estimate the dependence of $S_{i,\infty}$ on systemic inflammation index θ (panel A, dashed line and panel C).

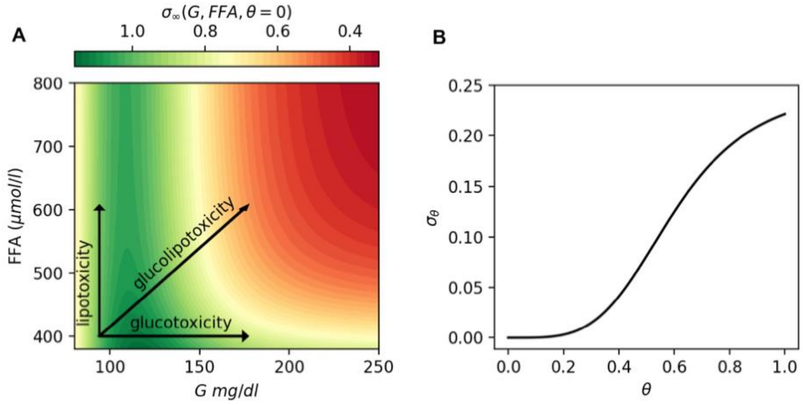

Figure S2: Dependence of σ_∞ on *G*, *FFA* and θ, related to STAR Methods. A) The $\theta=0$ cross-section of 3-dimensional σ_∞ manifold (Eq. 18) is color mapped on $G - FFA$ plane to illustrate the dependence of σ_∞ on G and *FFA*. B) σ_θ is an increasing sigmoid function of θ. Increasing G while keeping FFA at its baseline (Panel A, horizontal arrow), initially increases σ_∞, whereas an increase above 115 mg/dl causes a net decline in σ_∞, which signifies glucotoxicity. An increase in FFA while keeping G at its baseline (Panel A, vertical arrow), does not cause a significant effect. However, an increase in FFA for higher levels of G causes a substantial decline in σ_∞ due to glucolipotoxicity.

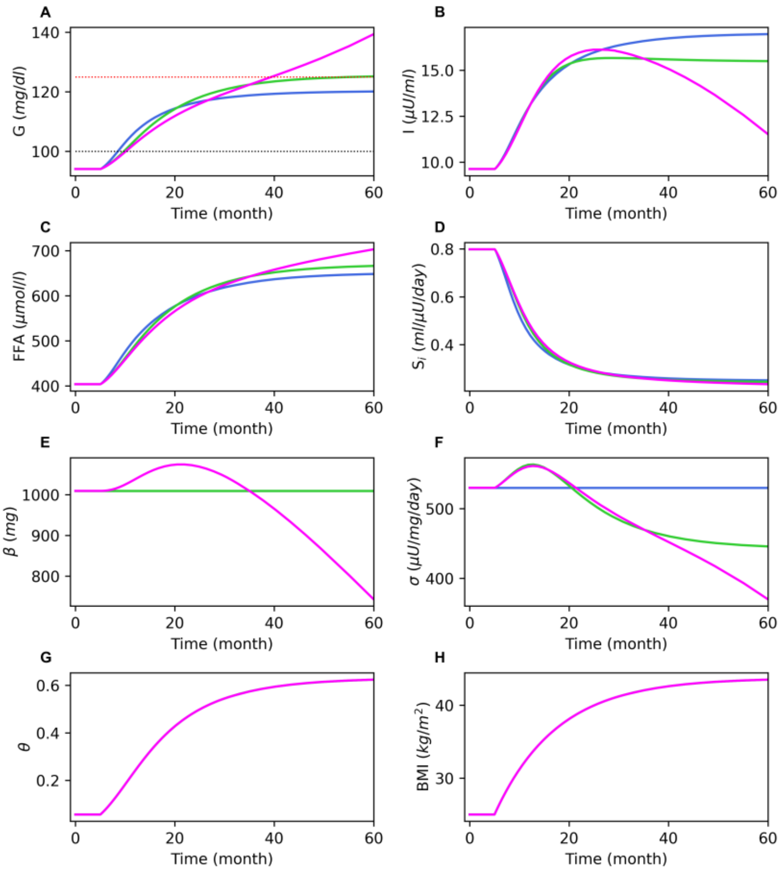

Figure S3: Decline in β-cell mass is necessary for overt diabetes, related to Figure 2. At T=5 months, DE_i is increased by 75%. For blue simulations, σ and β are fixed to their respective baseline values; for green simulations, only β is held fixed; for magenta simulations, all variables are let dynamically respond to the weight gain. In panel E, green and blue time courses overlap since β is fixed to its baseline value. In panels G and H, all time courses overlap, since weight gain and inflammation are not affected by σ or β. Simulations show that the decline in S_i is sufficient (Panel D, blue) to raise G (Panel A, blue) to the prediabetes ranges but not to the T2D levels when σ and β are held constant (Panels EF, blue). When decline in S_i is accompanied by a substantial decline in σ, while β is fixed, G reaches the T2D level (green). Although, the model shows that a substantial decline in β is necessary for the development of overt diabetes (Panel A, magenta), a more dramatic decline in σ that is caused by extremely high inflammation (θ>0.8) may also increase glucose above the diabetic ranges (135 mg/dl) without a decline in β (not shown).

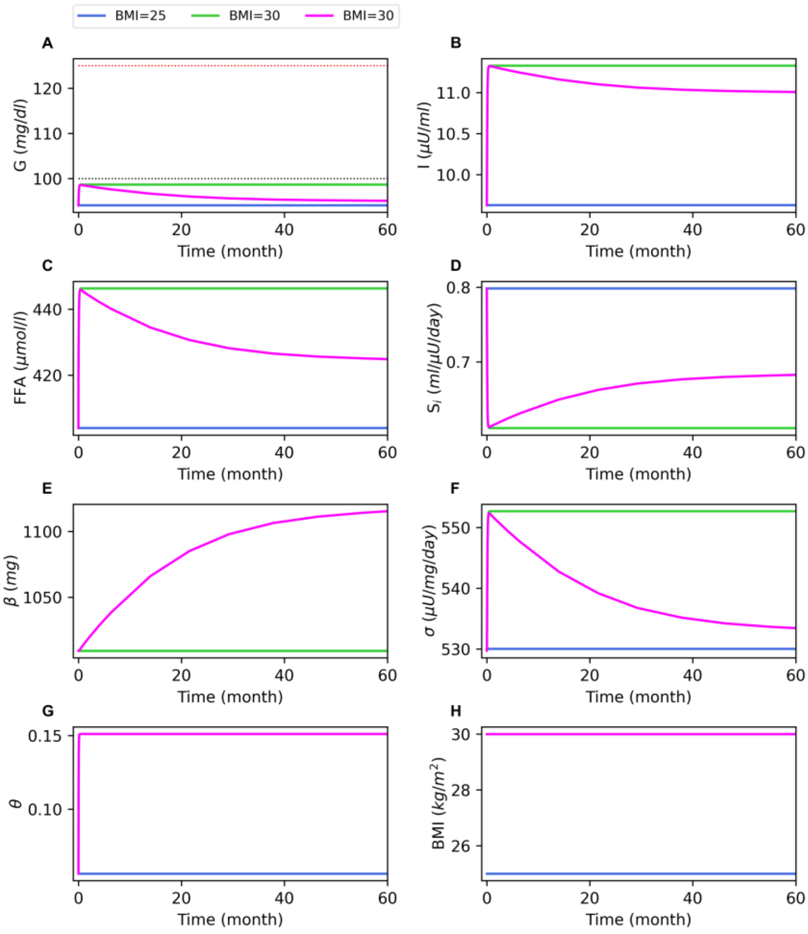

Figure S4: Slow vs. Fast variables of the system, related to Figure 3. The dynamic response of variables to the changes in BMI are shown. At T=0, the BMI is increased from 25 (blue) to 30 (green and magenta) *kg/m*^{2}. In green simulations, β is fixed to its baseline value, whereas, in magenta simulations it is let to dynamically respond to the weight change. In panel E, blue and green time courses overlap, since β remains at the baseline for these simulations, whereas in panels G and H, green and magenta time-courses overlap. The blue time-course shows that the system is at rest at a steady state with a BMI value at 25 *kg/m*^{2}. Setting BMI to 30 *kg/m*^{2}, while holding the slow variable β fixed at its baseline value, the fast (G and I) and intermediate (*FFA*, S_i, θ and σ) time-scale variables abruptly assume their corresponding steady-state values (green). Setting BMI to 30 *kg/m*^{2} and letting β to vary dynamically in response (magenta), the fast and intermediate time-scale variables quickly assume their steady state values and follow the β dynamics.

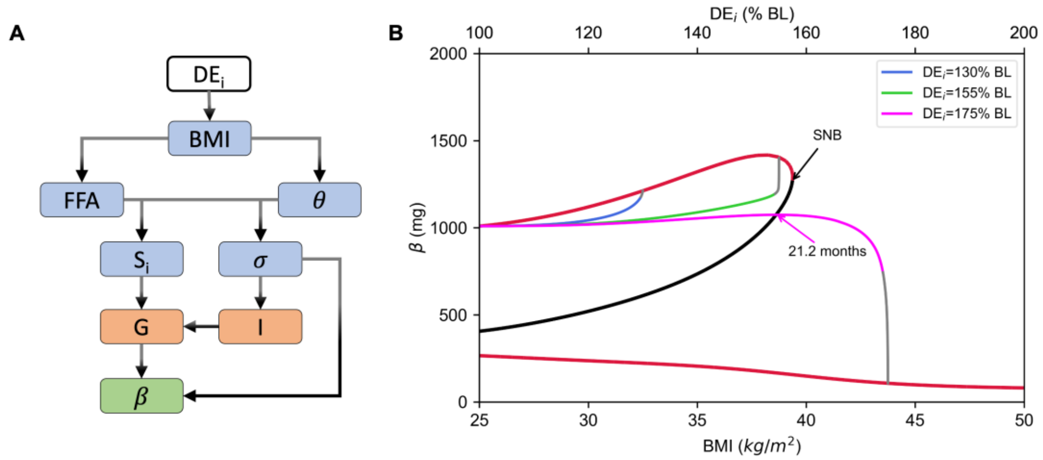

Figure S5: Continuation analysis of the system, related to Figure 3. A) Simplified model diagram that shows the propagation of a perturbation caused by a change in daily energy intake (DE_i) through the system. The timescales of the variables are color coded; orange: fast timescale, blue: intermediate timescale, green: slow timescale. In the model, DE_i only directly affects BMI. Hence, a perturbation caused by a change in DE_i propagates through the system via BMI. Hence, we use BMI as a parameter of the system, and calculate the steady-state solutions of the downstream variables for BMI values between 25-50 kg/m^2. B) Steady state solutions or the bifurcation diagram of the system is projected onto the BMI-β axes. Solid red curves show stable steady states, whereas solid black curve shows unstable steady sates. The superimposed trajectories are the projection of the solution curves given in Figure 2 in main text. The grey segments of the trajectories represent the portion of the corresponding solution curve that exceeds 60 months. The arrow labelled with 21.2 months indicate the point at which magenta solution takes its maximal β value in months, which coincides with the branch of unstable steady states. DE_i relative to the baseline that would result in the corresponding steady state BMI values are labelled on the top axis. SNB: saddle node bifurcation. When there is a change or a perturbation in BMI, variables on similar timescales as BMI (*FFA*, S_i, θ and σ) and the variables on faster timescales (*G* and *I*) quickly assume their respective values, whereas β slowly changes over time due to its slow dynamics (Fig. S4). Therefore, when the system is perturbed, in 8-dimensional phase space, the solutions move quickly in the directions of the fast and intermediate timescale variables (almost perpendicular to the β-axis) onto the stable slow manifold constituted by β-null surface. Then, the solutions slowly follow β dynamics and converge to a steady state (Fig. S4). Since the solutions quickly move almost perpendicular to the β-axis from all directions, and follow the slow β dynamics thereon, the projection of the 8-dimensional bifurcation diagram onto the *BMI*-β-axes captures the slow dynamics of the system accurately.

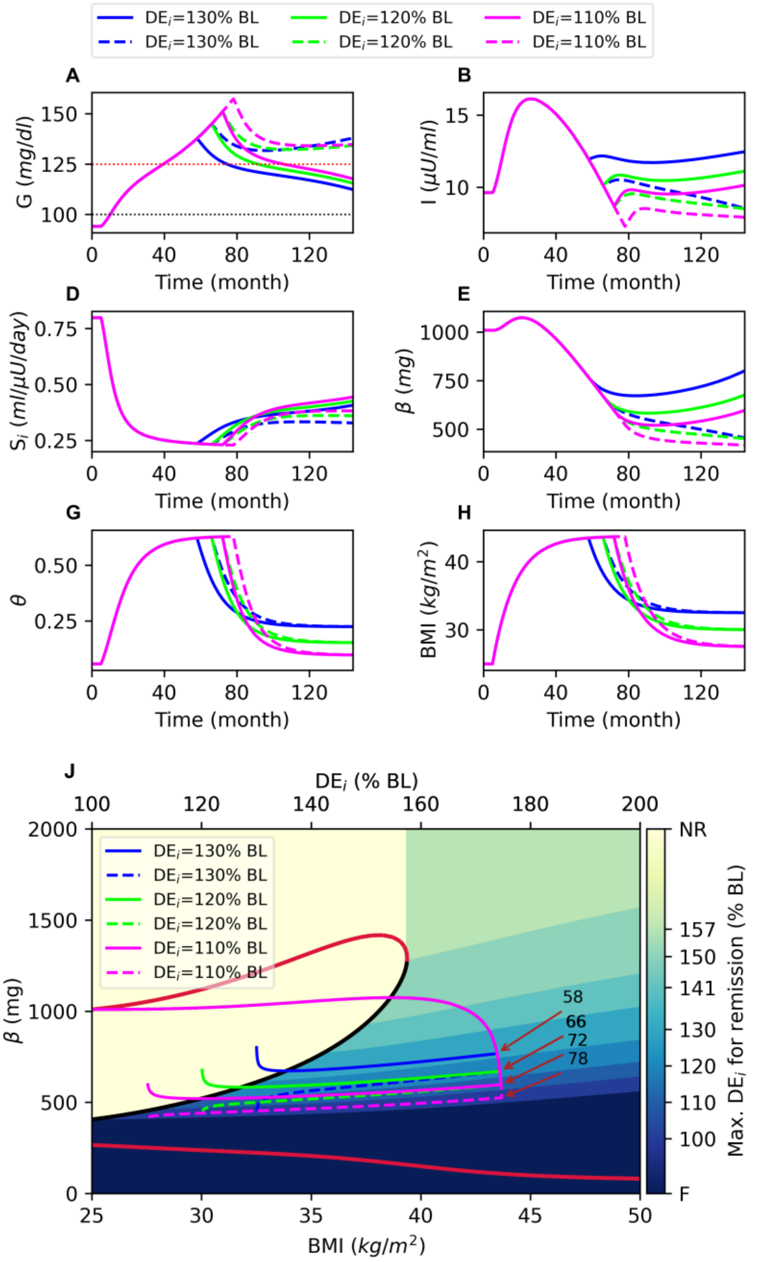

Figure S6: Diabetes duration and intensity of the calory restriction, related to Figure 4. At T=5 months, daily energy intake (*DE$_i$*) is increased by 75%, and it is set back to the 130% (blue), 120% (green) or 110% (magenta) of the baseline at T=58, 66, 72 or 78 months. A) Plasma glucose with prediabetes (black dotted line, 100 *mg/dl*) and diabetes cutoffs (red dotted line, 125 *mg/dl*). B) Plasma insulin, C) plasma FFA, D) insulin sensitivity, E) β-cell mass, F) β-cell function, G) inflammation index and H) BMI time courses. I) The disease progression index (DP) at every 24 months, where dashed time courses are indicated with hatched bars. J) The projection of the continuation diagram onto *BMI-β* axes with superimposed trajectories. The brown arrows are used to label the time points of the intervention. The daily energy intake (DE$_i$) relative to baseline that would result in the corresponding steady state BMI values are labelled at the top axis. The color bar shows the *DE$_i$* relative to baseline that is necessary for successful remission. NR: no restriction; F: failure. For blue trajectories, the daily energy intake is set to 130% of the baseline at T=58 (solid blue) and 66 months (dashed blue). At T=58 months, the phase point is within the region marked by 130% BL (Fig. S6J). Therefore, reducing *DE$_i$* to 130% of the baseline results in successful remission (solid blue). At T=66 months, the phase point reaches the region marked by 120% BL (Panel J). Therefore, reducing *DE$_i$* to 130% BL is not sufficient for remission (dashed blue). In this region, the *DE$_i$* must be reduced at least to 120% of the baseline for successful remission. Reducing *DE$_i$* to 120% BL at T=66 months results in successful remission (solid green). The same behavior is evident for solid and dashed magenta trajectories.

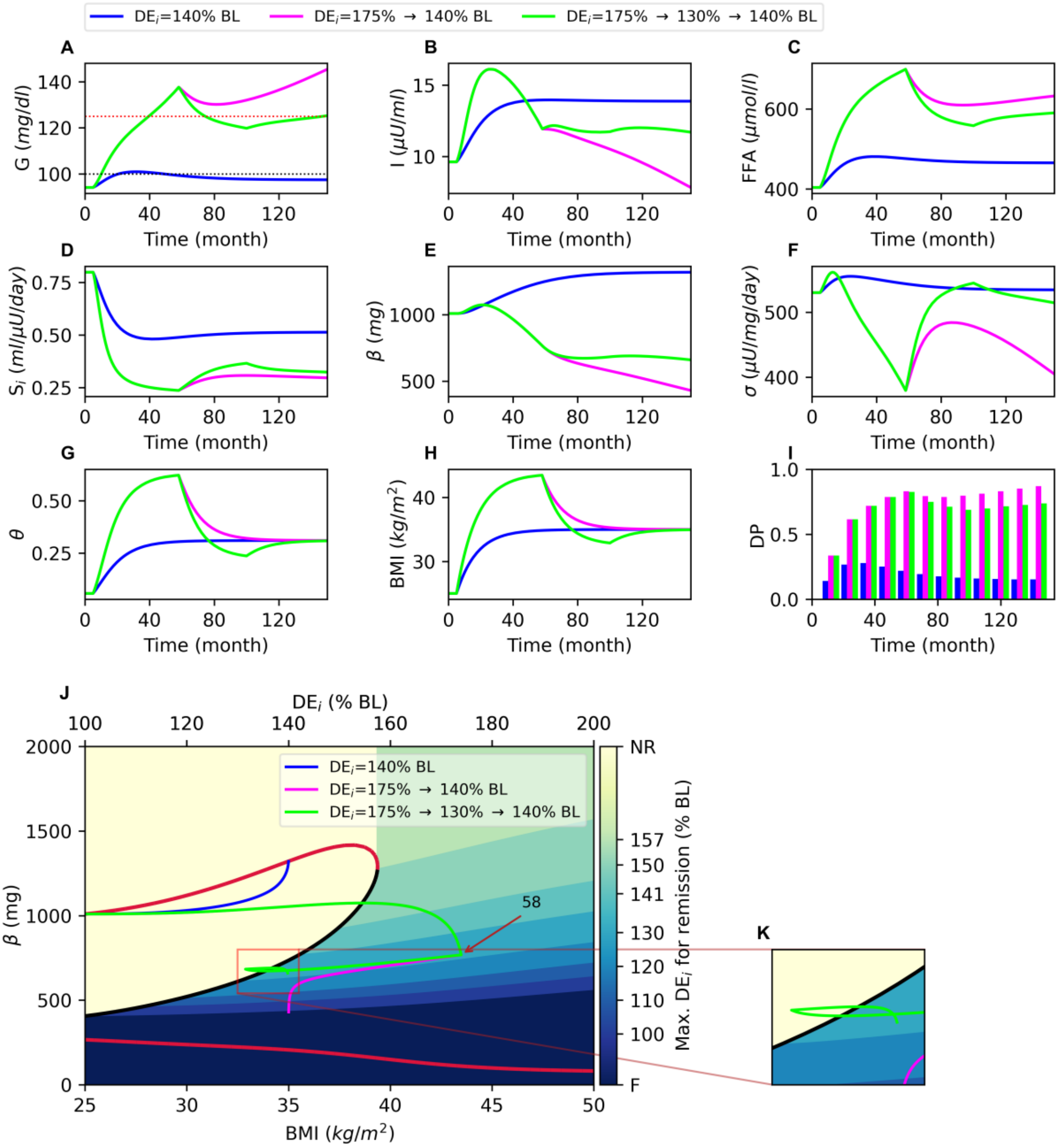

Figure S7: Diabetes can easily relapse after remission, related to Figure 4. At T=5 months, daily energy intake (DE_i) is increased to 140% (blue) or 175% (magenta and green) of the baseline (BL). At T=58 months, DE_i is reduced to 140% (magenta) and 130% (green) of the BL. At T=100 months, DE_i is increased again to 140% of the BL (green). A) Plasma glucose with prediabetes (black dotted line, 100 *mg/dl*) and diabetes cutoffs (red dotted line, 125 *mg/dl*). B) Plasma insulin, C) plasma FFA, D) insulin sensitivity, E) β-cell mass, F) β-cell function, G) inflammation index and H) BMI time courses. I) Disease progression index (DP) at every 12 months. J) The projection of the bifurcation diagram onto *BMI*-β axes with superimposed trajectories. DE_i relative to baseline for the corresponding steady state BMI values are labelled on the top axis. The color bar shows the maximum DE_i levels relative to baseline (% BL) necessary for successful remission for corresponding region. K) The region indicated by the red box is magnified. BL: baseline; NR: no restriction; F: failure. Prevention of diabetes is easier than achieving remission through weight loss. Once the trajectory passes to the right side of the threshold (Panel J) β starts to decline. As a consequence, the trajectory must travel a longer distance to the left to pass to the left side of the threshold (Panel J). After the remission, subject remains prone to relapse of diabetes for a long period of time. Because β has slow dynamics and it takes a significant amount of time for it to recover. Therefore, even a small perturbation caused by a relatively small weight gain can push trajectory back to the right side of the threshold (Panel J, green). A greater weight loss reduces the chances of relapse after remission.

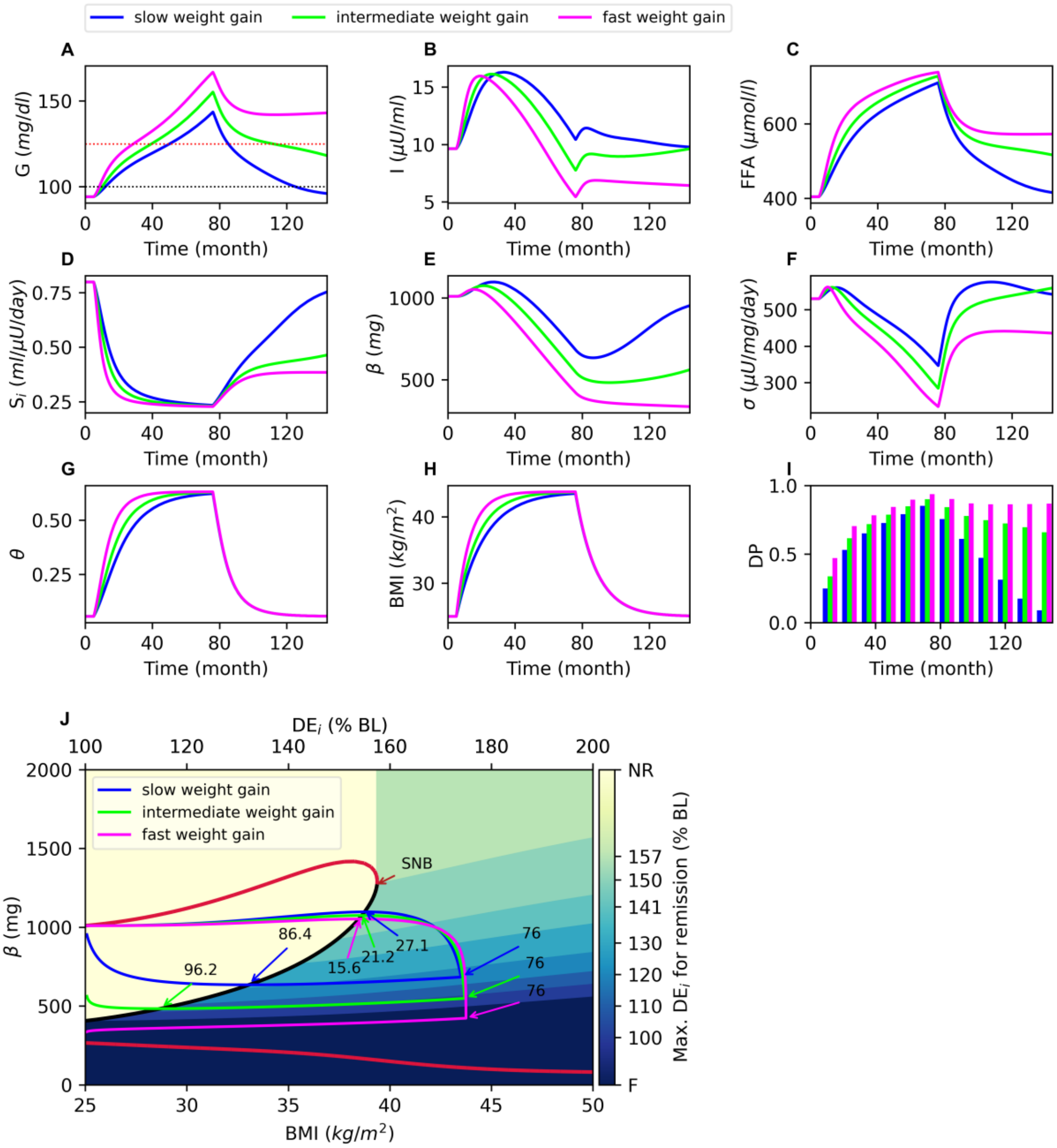

Figure S8: The pace of the weight gain determines success of the remission, related to Figure 4. At T=5 months, daily energy intake (*DE_i*) is increased to 175% of the baseline (BL) to simulate weight gain with different paces. *DE_i* is set back to the baseline value at T=76 months to simulate diet intervention. Figure S8 shows simulations for slow (blue), intermediate (green) and fast (magenta) weight gain rates, where the rate of the weight gain is determined by scaling the weight gain dynamics by a constant (Eq. S6, τ_W). For intermediate weight gain rate, the time constant is left as 1, whereas for slow weight gain rate it is set to 1.33 and for fast weight gain rate it is set to 0.67. For each scenario, after a period of weight gain phase of 76 months, we start a phase of diet intervention. In the intervention phase, τ_W is set to the 1 for all simulations to ensure same weight loss dynamics. This way we only focus on the effect of the rate of weight gain on the disease progression. A) Plasma glucose with prediabetes (black dotted line, 100 *mg/dl*) and diabetes cutoffs (red dotted line, 125 *mg/dl*). B) Plasma insulin, C) plasma FFA, D) insulin sensitivity, E) β-cell mass, F) β-cell function, G) inflammation index and H) BMI time courses. I) Disease progression index (DP) at every 12 months. J) The projection of the bifurcation diagram onto *BMI-β* axes with superimposed trajectories. The arrows are used to label the time points of certain events in months (β-maximum, intervention and β-minimum). DE_i relative to baseline for the corresponding steady state BMI values are labelled on the top axis. The color bar shows the maximum *DE_i* levels relative to baseline (% BL) necessary for successful remission for corresponding region. BL: baseline; NR: no restriction; F: failure; SNB: saddle node bifurcation.

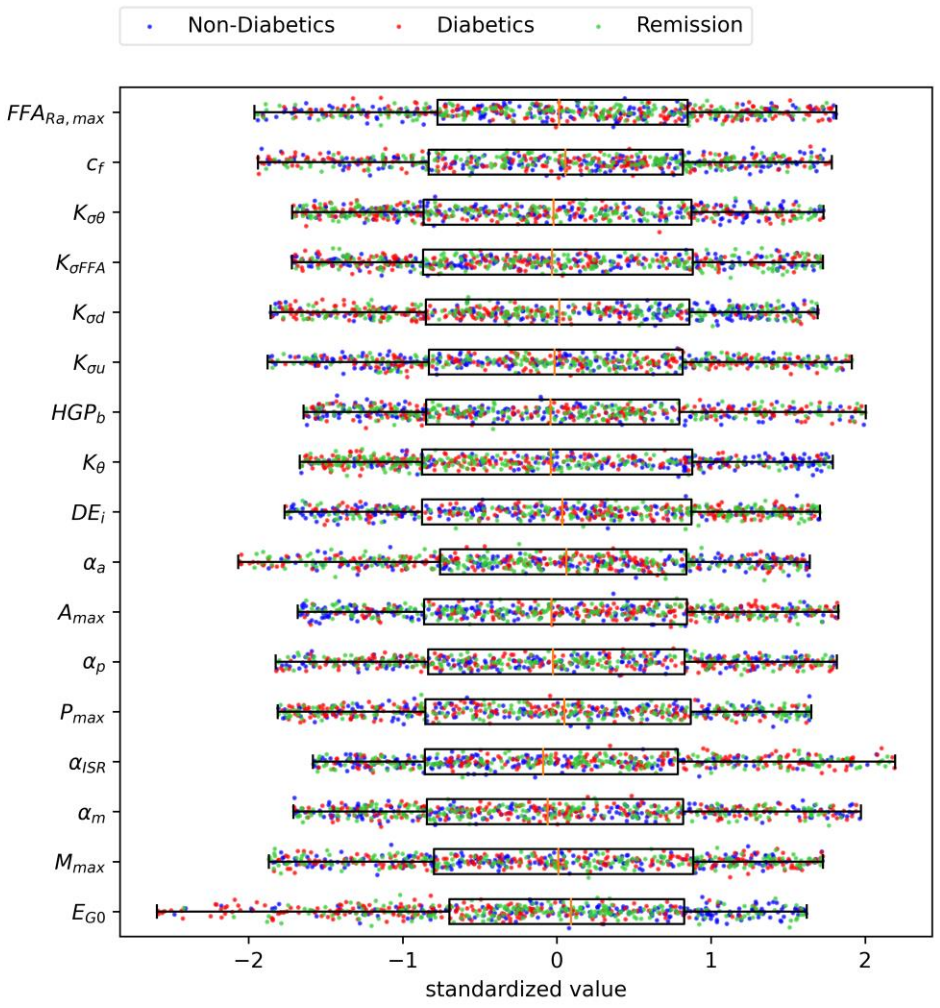

Figure S9: Distribution of the randomly sampled model parameters, related to Figure 6 and 7. Distributions of standardized values of the parameters used for generating virtual populations shown in Fig. 6, 7 and S10. Population simulations were generated by randomly sampling a subset of parameters shown. The subset of the parameters was selected based on a local sensitivity analysis, where each parameter was varied between ±%10 of its default value (Table S3), and the subset of the parameters resulted in the largest variation were selected for further analysis. For each parameter, 100,000 random values were selected from a uniform distribution defined between ±20% range of the values given in Table S1. Only DE_i was selected differently, where it was randomly selected from a uniform distribution defined between [150%,180%] of the baseline value. With this setup, 100,000 simulations were generated. Afterwards, simulations that resulted in physiological baseline values were selected for further analysis. The following ranges were used for physiological baseline values; G: 70-100 mg/dl; I: 8-12 μU/ml; FFA: 300-500 μmol/l; S_i: 0.7-0.9 ml/μU/day; β: 800-1200 mg; σ: 400-600 μU/mg/day. These constraints resulted in more than 3000 simulations in total. For each subgroup (Non-Diabetic, Diabetic and Remission), 200 simulations were randomly selected for illustration purposes.

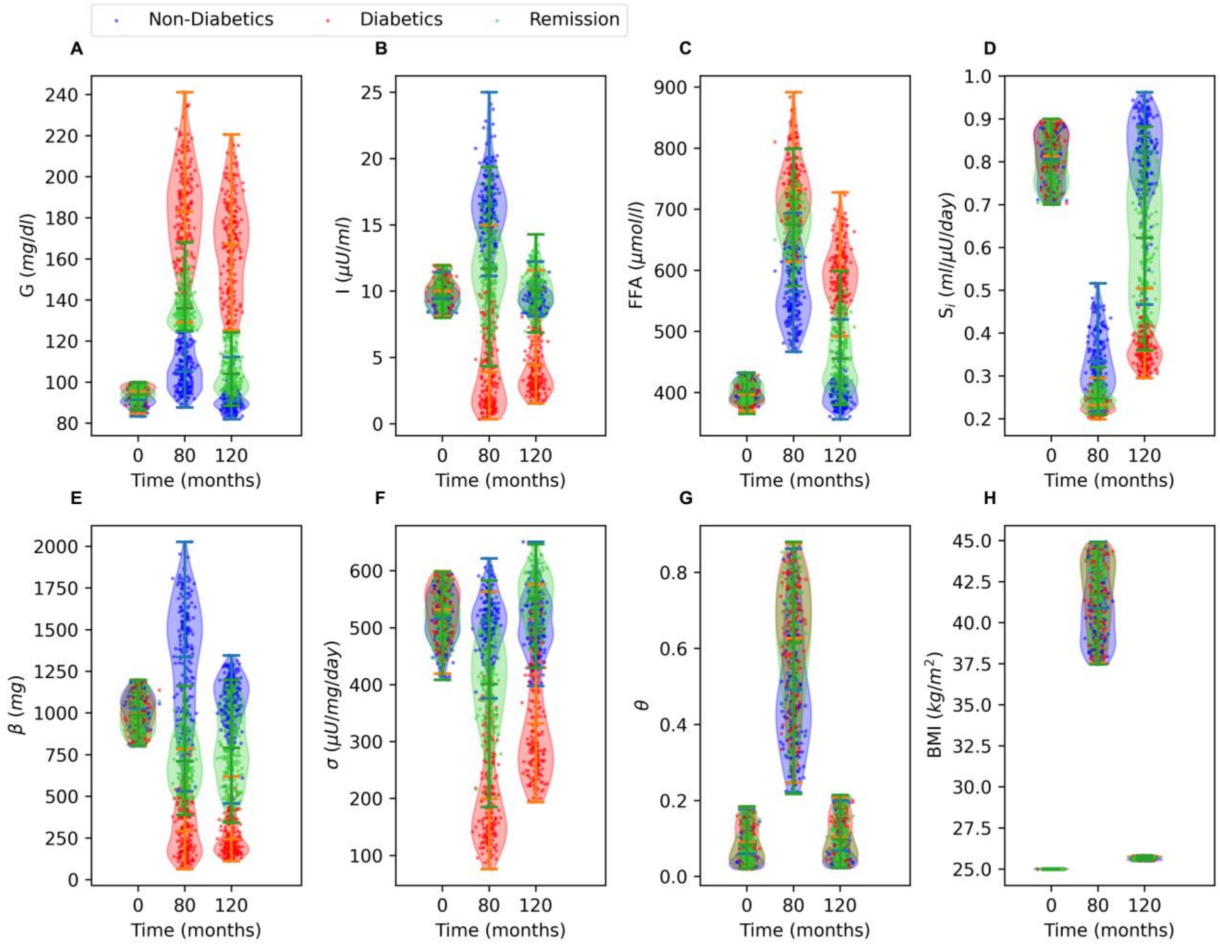

Figure S10: Distribution of virtual population at different time points, related to Figure 6 and 7. Violin plots of the model variables with individual data points at the beginning (T=0), at the intervention time point (T=80 months), and at the end of the simulations (T=120 months) given in Figure 8 in main text. Non-diabetics are the subjects whose plasma glucose levels remain below the T2D cutoff (125 mg/dl) throughout the simulation timeframe. Diabetics are the subjects whose plasma glucose increases above the T2D cutoff level and remains in this range. Remission group comprises the subjects whose plasma glucose rises above the T2D cutoff level but falls under this cutoff after the intervention.

Table S1. List of supplemental equations and their explanations, related to STAR methods.

Equation	Explanation	Number
$\boldsymbol{\sigma_{gu}} = M_u \frac{\boldsymbol{G}^{nu}}{\boldsymbol{G}^{nu} + K_{\sigma u}^{nu}}$	σ_{gu} is defined as an increasing sigmoid function of G and determines glucose dependent increase in σ during the early stages of IR.	**S1**
$\boldsymbol{\sigma_{gd}} = M_d \frac{\boldsymbol{G}^{nd}}{\boldsymbol{G}^{nd} + K_{\sigma d}^{nd}}$	σ_{gd} is a decreasing sigmoid function of G, and it represents the cytotoxic effects of glucose on σ.	**S2**
$\boldsymbol{\sigma_{FFA}} = M_{\sigma FFA} \frac{\boldsymbol{FFA}^{nFFA}}{\boldsymbol{FFA}^{nFFA} + K_{\sigma FFA}^{nFFA}}$	σ_{FFA} is a decreasing sigmoid function of FFA, and it represents the cytotoxic effects of FFA on σ.	**S3**
$\boldsymbol{\sigma_{\theta}} = M_\theta \frac{\boldsymbol{\theta}^{n\theta}}{\boldsymbol{\theta}^{n\theta} + K_{\sigma\theta}^{n\theta}}$	σ_θ is an increasing sigmoid function of θ, and accounts for the detrimental effect of inflammation on σ.	**S4**
$\boldsymbol{FMR} = F_b \boldsymbol{BMI} + F_{age} Age + F_0$	Fat mass ratio (*FMR*) is calculated from BMI and age using the formulation proposed before [1]. The baseline age is used as input and the age change during the simulation timeframe is ignored, since the change in age has a relatively small effect compared to BMI within the simulation timeframe.	**S5**
$\frac{d\boldsymbol{W}}{dt} = \frac{k_W \cdot DE_i - DE_e \cdot \boldsymbol{W}}{\tau_W}$	Weight gain dynamics are scaled for Figure S8 by τ_W.	**S6**

Table S2: Model variables and baseline values, related to STAR Methods.

Variable	Initial Value	Explanation
W	81 kg	Body weight
G	94 mg/dl	Plasma glucose concentration
I	9.6 µU/ml	Plasma insulin concentration
FFA	400 µmol/l	Plasma FFA concentration
S_i	ml/µU/d	Insulin sensitivity
β	1000 mg	β-cell mass
σ	530 µU/mg/d	β-cell function
θ	0.05	Systemic inflammation index

Table S3: Model parameters and their physiological explanations, related to STAR Methods and Figure 2.

Parameter	Value	Explanation	Ref.
E_{G0}	24.48 d^{-1}	Glucose effectiveness	[6,7]
HGP_b	2000 mg/dl/d	Insulin independent basal hepatic glucose production	[6,7]
$HEPA_{max}$	3000 mg/dl	Insulin dependent component of maximal hepatic glucose production	[6,7]
α_{HGP}	4 d^{-1}	Half maximal value for hepatic insulin action	[6,7]
$HEPA_{Si}$	ml/µu/d	Hepatic insulin sensitivity. (Set to S_i)	[8]
V	5 lt	Plasma volume	
k	700 d^{-1}	Insulin excursion rate	[2]
K_{ISR}	2	Hill coefficient for ISR	[2]
α_{ISR}	1.2	M level for half maximal ISR	[2]
K_M	2	Hill coefficient for M	[2]
α_M	140	G level for half maximal M	[2]
α_{SIF}	2	Hill coefficient for insulin dependent suppression of FFA production	[9]
K_{SIF}	11 d^{-1}	Half maximal insulin effectiveness ($S_i.I$) for lipolysis suppression	[9]
c_f	2 $kg^{-1}d^{-1}$	FFA clearance rate per kg body weight	[9]
F_{Ra0}	188	Intercept for the linear relation between FM and FFA_{Ra}	[10]
F_{Ra1}	8.96	Slope for the linear relation between FM and FFA_{Ra}	[10]

k_w	8.7e-5 kg/cal	kg weight gain per calorie. The value is set to the 2/3 of the value published in [11] to accommodate slower weight gain per calorie intake in accordance with [12].	[11,12]
DE_i	2500 cal/d	Total daily energy intake	[11]
W_b	81 kg	Body weight at the baseline	
h	1.8 m	Height (fixed)	
Age	30	Age at the baseline.	
DE_e	1.07e-6 d^{-1}	The rate of daily weight loss due to energy expenditure, at 0 calorie intake. Calculated from the steady state baseline body weight using the baseline DE_i.	
τ_W	1	Weight gain time constant	
τ_θ	1 d	Inflammation time constant	
n_θ	6	Hill coefficient for θ_∞	
K_θ	40 kg/m^2	BMI value for half maximal inflammation response	
τ_{Si}	1 d	Insulin sensitivity time constant	
$S_{i,b}$	1.4 ml/μu/d	Asymptotic value for $S_{i,\infty}$ at zero FFA and θ	[3]
M_{FFA}	0.8	Maximal dependence of $S_{i,\infty}$ on FFA.	[3]
n_{Si}	1	Hill coefficient for $S_{i,\infty}$ response to FFA.	[3]
$K_{Si,FFA}$	400 μmol/l	FFA level for half maximal $S_{i,\infty}$.	[3]
$K_{Si,\theta}$	1.8	$S_{i,\infty}$ responsiveness parameter to inflammation	[4]
P_{ng}	350 mg/ τ_β	β-cell neogenesis rate (fitted)	
s	0.0002 mg^{-2} τ_β^{-1}	Pairwise interaction/competition rate (fitted)	
τ_β	1800 d	β-cell mass time constant	[2]
τ_σ	1 d	β-cell function time constant	
σ_b	536 mU/mg/d	Baseline β-cell function	[2]
M_u	1.5	Maximal value for σ_u	[2]
n_u	6	Hill coefficient for σ_u	[2]
$K_{\sigma u}$	81 mg/dl	Glucose level for half maximal value for σ_u	[2]
M_d	1	Maximal value for σ_d	[2]
n_d	6	Hill coefficient for σ_d	[2]
$K_{\sigma d}$	137 mg/dl	Glucose level for half maximal steady state value for σ_d	[2]
$M_{\sigma FFA}$	1.2	Maximal value for σ_{FFA} (fitted)	
n_{FFA}	6	Hill coefficient for σ_{FFA} (fitted)	
$K_{\sigma FFA}$	357 μmol/l	FFA level for half maximal steady state value for σ_{FFA} (fitted)	
M_θ	0.25	Maximal value for σ_θ (fitted)	
n_θ	6	Hill coefficient for σ_θ (fitted)	
$K_{\sigma\theta}$	0.6	θ value for half maximal response in σ_θ (fitted)	
P_{max}	4.55	Maximal β-cell replication rate	[2]
α_P	35 μU/ml/d	ISR for half maximal β-cell replication rate	[2]
n_P	4	Hill coefficient for β-cell replication rate	[2]
A_b	0.8	M independent β-cell apoptosis rate	[2]
A_{max}	5	Maximal M dependent β-cell apoptosis rate	[2]
α_A	0.37	M level for half maximal β-cell apoptosis rate	[2]
n_A	6	Hill coefficient for β-cell apoptosis rate	[2]
F_b	1.25	Slope for FMR vs BMI	[1]
F_{age}	0.23	Slope for FMR vs Age	[1]
F_0	-16.2	Intercept for FMR vs BMI	[1]

Table S4: DIRECT Study data from Mrabeh et al., 2020, [13]**, related to Figures 8 and 9.**

	Time (months)	Glucose (mmol/l)	Insulin (pmol/l)	CRP (mg/l)	BMI (kg/m2)	Baseline Age
**Responders**	0	8±2,4	85(55,5-143,5)	2,4(1-5,4)	34,8±4,4	53.2±7.3
	5	5,7±0,8	31(19,5-52,3)	---	29,3±4,1	--
	12	5,8±0,9	34,3(19,1-50)	0,9(0,4-1,8)	30,1±4,6	--
	24	6,6±1,9	46,2(18,5-82,3)	1,3(0,4-4,3)	31,8±4,9	--
**Non-Responders**	0	9,4±2,9	65,5(48,2-86,9)	3,4(1,4-4,1)	34,7±4,6	52.8±8.2
	5	8,9±2,7	29(19,6-38,8)	---	30,04±4,8	--
	12	8,4±1,9	28(23,2-47,7)	1,5(1-1,8)	31,1±4,7	--
	24	9,3±4	34,4(16,5-49,5)	1,6(0,4-2,2)	31,7±4,5	--

The data is given as mean ± standard deviation or median(inter quartile range (IQR)) for responders (top rows) and for non-responders (bottom rows). CRP data was not recorded during the 5th month follow-up for either group. Hence, this data is extrapolated from the measurements recorded at 12th and 24th months using linear extrapolation. CRP and fasting insulin data were published as median(IQR). For insulin and CRP data, mean and sd are estimated as described before [14]. The glucose data unit is converted from mmol/L to mg/dl and insulin data unit is converted from pmol/L to μU/ml. To compare the CRP time course to the model estimate for systemic inflammation level (θ), we constrained CRP levels between [0,1] by scaling over 6 mg/L (2 times the cut-off value for low grade systemic inflammation [15]).

Table S5: Parameter table for DIRECT study data, related to Figure 8 and 9.

Parameter	Unit	Responders	Non-Responders
$HEPA_{max}$	mg/dl	3128.7 ± 655.9	3535.1 ± 9355.2
K_{θ}	kg/m^2	34.95 ± 2.39	34.92 ± 1.49
$S_{i,b}$	ml/mu/d	2.07 ± 3.47	1.97 ± 15.69
$K_{si,FFA}$	mmol/l	445.8 ± 358.8	424.63 ± 1305.65
$K_{si,\theta}$	---	0.45 ± 0.12	0.44 ± 0.39
τ_{σ}	d	698 ± 856.9	502.7 ± 1285.6
$Inc.\ DE_{i,5}$	% + BL	38.1 ± 4.3	41.2 ± 20.3
$Inc.\ DE_{i,60}$	% + BL	-30.2 ± 22.1	-23.1 ± 30.4
$Inc.\ DE_{i,65}$	% + BL	30	30
n_{si}	---	6	6
K	d^{-1}	900	900

The parameter values are estimated using DIRECT study data for responders and non-responders given in Table S4. Estimated parameter values are reported as estimated value ± confidence intervals, whereas for the fixed parameters the same value is reported for both groups.

References

1. Deurenberg, P., Weststrate, J.A., and Seidell, J.C. (1991). Body mass index as a measure of body fatness: age- and sex-specific prediction formulas. British Journal of Nutrition *65*, 105–114. 10.1079/BJN19910073.
2. Ha, J., Satin, L.S., and Sherman, A.S. (2016). A mathematical model of the pathogenesis, prevention, and reversal of type 2 diabetes. Endocrinology *157*, 624–635. 10.1210/en.2015-1564.
3. Boden, G., Chen, X., Ruiz, J., White, J. V., and Rossetti, L. (1994). Mechanisms of fatty acid-induced inhibition of glucose uptake. Journal of Clinical Investigation *93*, 2438–2446. 10.1172/JCI117252.
4. Recasens, M., López-Bermejo, A., Ricart, W., Vendrell, J., Casamitjana, R., and Fernández-Real, J.M. (2005). An inflammation score is better associated with basal than stimulated surrogate indexes of insulin resistance. Journal of Clinical Endocrinology and Metabolism *90*, 112–116. 10.1210/jc.2004-0708.
5. Doke, J. (2023). GRABIT - File Exchange - MATLAB Central. https://nl.mathworks.com/matlabcentral/fileexchange/7173-grabit.
6. Ha, J., and Sherman, A. (2020). Type 2 diabetes: one disease, many pathways. Am J Physiol Endocrinol Metab *319*, E410–E426. 10.1152/ajpendo.00512.2019.
7. Kahn, S.E., Prigeon, R.L., McCulloch, D.K., Boyko, E.J., Bergman, R.N., Schwartz, M.W., Neifing, J.L., Ward, W.K., Beard, J.C., Palmer, J.P., et al. (1994). The contribution of insulin-dependent and insulin-independent glucose uptake to intravenous glucose tolerance in healthy human subjects. Diabetes *43*, 587–592. 10.2337/diab.43.4.587.
8. Campbell, P.J., Mandarino, L.J., and Gerich, J.E. (1988). Quantification of the relative impairment in actions of insulin on hepatic glucose production and peripheral glucose uptake in non-insulin-dependent diabetes mellitus. Metabolism *37*, 15–21. 10.1016/0026-0495(88)90023-6.
9. Periwal, V., Chow, C.C., Bergman, R.N., Ricks, M., Vega, G.L., and Sumner, A.E. (2008). Evaluation of quantitative models of the effect of insulin on lipolysis and glucose disposal. Am J Physiol Regul Integr Comp Physiol *295*, 1089–1096. 10.1152/ajpregu.90426.2008.
10. Mittendorfer, B., Magkos, F., Fabbrini, E., Mohammed, B.S., and Klein, S. (2009). Relationship between body fat mass and free fatty acid kinetics in men and women. Obesity *17*, 1872–1877. 10.1038/oby.2009.224.
11. Crielaard, L., Dutta, P., Quax, R., Nicolaou, M., Merabet, N., Stronks, K., and Sloot, P.M.A. (2020). Social norms and obesity prevalence: From cohort to system dynamics models. Obesity Reviews *21*, 1–17. 10.1111/obr.13044.
12. Chow, C.C., and Hall, K.D. (2008). The Dynamics of Human Body Weight Change. PLoS Comput Biol *4*, e1000045. 10.1371/JOURNAL.PCBI.1000045.
13. Al-Mrabeh, A., Hollingsworth, K.G., Shaw, J.A.M., McConnachie, A., Sattar, N., Lean, M.E.J., and Taylor, R. (2020). 2-year remission of type 2 diabetes and pancreas morphology: a post-hoc analysis of the DiRECT open-label, cluster-randomised trial. Lancet Diabetes Endocrinol *8*, 939–948. 10.1016/S2213-8587(20)30303-X.
14. Wan, X., Wang, W., Liu, J., and Tong, T. (2014). Estimating the sample mean and standard deviation from the sample size, median, range and/or interquartile range. BMC Med Res Methodol *14*, 1–13. 10.1186/1471-2288-14-135/TABLES/3.
15. Osimo, E.F., Baxter, L.J., Lewis, G., Jones, P.B., and Khandaker, G.M. (2019). Prevalence of low-grade inflammation in depression: A systematic review and meta-Analysis of CRP levels. Psychol Med *49*, 1958–1970. 10.1017/S0033291719001454.
